# Insights into the influence of dog and guardian demographics, nutrition, and relationship on raw feeding practices

**DOI:** 10.3389/fvets.2026.1793754

**Published:** 2026-05-20

**Authors:** Panagiotis Balousis, Sarah K. Abood, Lauren Brazeau, Deep Khosa, Adronie Verbrugghe

**Affiliations:** 1 Department of Clinical Studies, University of Guelph Ontario Veterinary College, Guelph, ON, Canada; 2 Department of Population Medicine, University of Guelph Ontario Veterinary College, Guelph, ON, Canada

**Keywords:** canine nutrition, guardian perceptions, human nutrition, human-animal bond, mixed raw diets, pet food selection, raw meat-based diets, survey

## Abstract

**Introduction:**

Raw meat-based diets (RMBD) are increasingly popular among dog guardians, yet the role of personal nutrition habits and pet-guardian relationships in their adoption remains unclear. This study examines how these factors, alongside previously identified drivers of pet food selection, specifically relate to the decision to feed RMBD.

**Methods:**

Data from 433 dog guardians were collected via an e-survey, from which 46 questions were selected for analysis in this study. Participants were placed into raw or cooked groups based on their dogs’ primary diet. Responses were assessed using descriptive statistics and univariate logistic regression.

**Results:**

A cooked diet was the primary feeding method for 291 participants, while 142 reported providing a diet that included at least one raw element. Households with an income exceeding CA $100,000 were predictive of raw feeding, while possession of graduate education was protective against it. Intact dogs of medium size were also associated with raw feeding practices. Compared to supermarkets and veterinary clinics, pet specialty stores were significantly more likely to be used by raw feeders for pet food purchases. Additionally, the provision of supplements to ensure dietary balance was linked to raw feeders, with the inclusion of fish oil increasing the odds of feeding RMBD. Raw feeding was also associated with greater perceived knowledge of canine nutrition and a preference for minimally processed pet foods, whereas emphasis on the convenience or cost of feeding reduced the likelihood of adopting RMBD. Survey questions evaluating guardians’ nutrition habits and perceived relationships with their pets did not predict diet group membership.

**Conclusion:**

Differences between raw feeders and guardians feeding cooked diets were observed in demographics (e.g., income and education), dog characteristics (e.g., breed size and sexual status), supplement use, pet food opinions and purchasing locations, and perceived dog nutrition knowledge. However, no differences were found in personal nutrition habits or the perceived human-animal bond.

## 1 Introduction

The growing trend of anthropomorphizing companion animals strengthens the emotional bond between pets and guardians, fostering greater concern for pets’ well-being and encouraging more attention to the quality of their diet and overall eating experience (1). The feeding of raw meat-based diets (RMBD) has gained popularity among guardians in Western nations who are dissatisfied with traditional kibble and wet pet foods (2–4). The rising interest in raw feeding may stem from its perceived benefits, as guardians report superior digestibility and improved skin, coat, and dental conditions (4–6). However, RMBD have been criticized for potential nutritional imbalances and increased risks of microbial contamination (4, 5, 7–10). Therefore, the growing popularity of RMBD highlights the importance of understanding the motivations driving the adoption of these practices.

Pet food selection is multifactorial, with the choice being strongly influenced by pet guardians’ views and preferences on feeding practices. Natural ingredients are viewed as a marker of quality in pet foods (5, 6, 11–13). As a result, commercial manufacturers of cooked diets (i.e., kibble and wet foods) are doubted in their ability to provide proper nutrition, due to concerns over the use of processed ingredients and artificial additives (3, 4). Specifically, guardians may perceive conventional diets as posing health risk for their pets, with surveys indicating that processed and artificial ingredients in diets are thought to compromise pet welfare (3, 11, 12). While the presence of unprocessed ingredients may contribute to the appeal of raw diets, additional factors influencing guardians’ feeding choices should be explored to better understand the motivations behind feeding RMBD.

In addition to ingredient preferences, guardians’ socioeconomic factors and personal eating habits could shape pet feeding routines. The price of raw diets can be a barrier in adopting such practice, though this concern may diminish for those with higher incomes (5, 14–16). Guardians who opt for natural, unprocessed foods for themselves may also be more likely to apply similar health-oriented principles to their pets’ diets (16, 17). As guardians develop closer emotional bonds with their pets, they may be more inclined to extend individual healthy eating habits to companion animals, reflecting a desire to support their pets’ well-being. Although pet guardians’ perceptions of their human-animal relationships and personal nutrition could affect pet feeding practices, additional research is needed to understand their impact on the adoption of raw diets.

Although regression models have been rarely applied in prior research profiling raw feeders, a survey of UK dog guardians relied on regression analyses to identify factors predictive of raw feeding, including dog breed, age, sexual status, and guardians’ feeding practices and views on pet diets (6). The objective of the present study was to expand the current understanding of RMBD selection by investigating previously identified factors influencing pet food selection and assessing their specific impact on the adoption of raw feeding practices using regression models. Factors examined included guardian demographics, dog characteristics and diets, guardians’ pet diet beliefs, dog food selection preferences, and perceived knowledge of dog nutrition. In addition, although guardians’ perceptions of the human-animal relationship and their personal dietary habits may influence pet feeding practices, these variables remain largely underexplored in studies investigating factors influencing raw diet adoption. Therefore, the study also aimed to examine guardians’ dietary practices and perceptions of the human-animal bond in relation to RMBD feeding. Based on prior research suggesting that pet guardians’ income, personal dietary habits, and feeding beliefs influence pet food choices, and that raw feeders report distinctive attitudes towards feeding practices, we hypothesized that guardians providing RMBD would differ from those selecting cooked diets across the factors explored in this study.

## 2 Materials and methods

Approval for the study was granted by the University of Guelph Research Ethics Board (REB #21–03-021), confirming compliance with ethical guidelines for human research. Before participation, all respondents provided informed consent electronically after reviewing a form describing the study’s objectives and the anonymous use of their data for research purposes.

### 2.1 Study design

The survey (see Supplementary material) was developed using Qualtrics (Qualtrics LLC, Provo, Utah, United States) and was accessible online from June to July 2022. The authors developed the questionnaire with input from the industry partner, and both parties conducted pilot testing to refine the survey questions. Recruitment relied on convenience sampling by advertising the survey on 15 non-nutrition, dog-related Facebook pages (Facebook Inc., Menlo Park, California, United States). Individuals responsible for more than one dog were instructed to focus their responses on the dog whose name started with the letter nearest to “A” in the English alphabet. To encourage participation, respondents could enter a prize draw to win one of four CA $30 Amazon gift cards. Email addresses were collected for the draw in a separate survey to maintain the anonymity of responses provided in the main questionnaire.

### 2.2 Survey design

The study was structured as part of a larger survey aimed at gathering information about choices and habits regarding raw pet food feeding practices among dog guardians. A total of 46 questions were analyzed for this study: 28 multiple-choice, 5 multi-select, 10 short-answer, and 3 slider-scale questions. The relevant questions collected information about pet guardian demographics and dog characteristics, as well as details on the dogs’ diets, the guardians’ dog food choices and perceived knowledge of dog nutrition, insights into their personal nutrition choices and self-reported knowledge of human nutrition, and views of their human-animal relationship.

Demographic data were collected through questions centered on the age, gender, residence, education level, employment, and household income of the respondents. To obtain information on dog characteristics, pet guardians were asked questions about their dog’s breed, age, sex, sexual status, acquisition source, activity level, the presence of other pets in the household, whether their dog lived indoors or outdoors, and how long the pet had been residing with the guardian. Information about the diet was primarily collected through a multi-select question where guardians could choose from a list of various feeding practices that reflected their dog’s nutrition-options included: extruded dry/kibble, wet/canned, fresh refrigerated steam-cooked, dehydrated or air-dried, fresh refrigerated raw, freeze-dried raw, frozen raw, homemade cooked, and homemade raw diets. Follow-up questions on dietary habits requested the location of pet food purchasing, the feeding routine for those including homemade elements, the duration for which the current diet had been fed, whether supplements were provided, supplement types, and the reasons for administering supplements. Moreover, participants were requested to discuss any dietary changes for their dog in the last 4–6 months and any anticipated changes in the coming 4–6 months. The guardians’ understanding and views of dog nutrition were assessed through questions regarding how often they thought about their dog’s nutrition and their self-perceived knowledge of canine nutrition. For this topic, slider questions were also used to understand participants’ perceptions of the extent to which their dog’s diet was “processed,” “preserved” or “raw,” their satisfaction with their dog’s current diet, and their agreement level with nine statements exploring beliefs and considerations about canine nutrition. A multi-select question featured 41 variables to assess factors influencing dog food choices. Additionally, the guardians’ nutrition choices and knowledge of human nutrition were examined primarily via multiple-choice questions centered on dietary habits and restrictions, involvement in household food preparation, weekly consumption of pre-made food, perception of their diets’ healthfulness and level of human nutrition knowledge, the frequency in which they thought about their personal nutrition, and their confidence in making self-directed dietary changes. Lastly, human-animal relationships were evaluated from the respondents’ description of the role and relationship of their dog in their lives.

### 2.3 Survey analysis

Prior to analysis, all participants were screened to confirm they met study inclusion criteria. Eligible participants were those who self-identified as the primary decision maker for their dog’s diet and reported the type of diet fed to their dog. Descriptive and inferential statistics were then carried out using SPSS (IBM Corp., version 29, Armonk, New York, United States). Partially completed surveys were retained in the dataset if participants met the inclusion criteria, so the number of responses varied across survey questions.

Response patterns across diet groups were examined using descriptive statistics, which included summarizing the number of responses (*n*) per question and the selection frequency (%) of response options. Short-answer responses were manually reviewed to identify recurring topics, with similar responses grouped to summarize key topic trends. Dog age was grouped into four categories: puppy (birth-12 months), young adult (13 months–23 months), mature adult (2 years–6 years), and senior (>6 years of age) (18). Breeds were categorized into groups based on the Canadian Kennel Club’s (CKC) seven breed groups: herding, hound, non-sporting, sporting, terrier, toy, and working dogs (19). A mixed category was also included for those who reported their dog as a cross of two or more breeds. Moreover, dogs were categorized by size based on the average weight range for each breed: small (<10 kg), medium (10–24 kg), large (25–40 kg), and giant (>40 kg) (20). Mixed breeds were excluded from grouping due to their unknown weight ranges. Regarding the presence of other pets in the household, multi-pet households included respondents with more than one dog or any additional pet residing with their canine companion, whereas single-pet households were those caring for only one dog. Responses from the multi-select question listing feeding practices were used to organize participants into one of six diet groups: commercial cooked (extruded dry/kibble, wet/canned, dehydrated or air-dried, and fresh refrigerated steam-cooked pet food), homemade cooked, mixed cooked (a combination of homemade and commercial cooked foods), commercial raw (fresh refrigerated, freeze-dried, and frozen raw pet food), homemade raw, or mixed raw (any raw feeding including a cooked component or a mix of commercial and homemade raw feeding). Participants were subsequently assigned to the cooked group (commercial, homemade, and mixed cooked) if their dog’s main diet contained only heat-processed items, or the raw group (commercial, homemade, and mixed raw) if the primary diet included at least one element that was not heat-processed. Slider-scale questions, scored from 0 to 100, were grouped into defined categories for analysis. Specifically, with respect to the question about the participants’ satisfaction levels with their current diet, responses were categorized into the following ranges: extremely dissatisfied (0–19), somewhat dissatisfied (20–39), neither satisfied nor dissatisfied (40–60), somewhat satisfied (61–80), extremely satisfied (81–100). The following categories were also created to group guardians’ perceptions of how processed, preserved, and raw they considered their dogs’ diets to be: very little (0–30), neutral (31–70), very much (71–100). Lastly, the responses from the nine statements exploring beliefs and considerations concerning canine nutrition were interpreted according to the following ranges: strongly disagree (0–30), neutral (31–70), strongly agree (71–100).

Inferential statistics were conducted using binomial logistic regression with a significance level of 0.05. Univariate analyses were performed for each survey question, with the diet groups (i.e., cooked or raw) acting as the dependent variable and survey questions serving as independent variables. In both descriptive and inferential statistics, after participants were categorized into their respective diet groups, homemade cooked feeders were omitted from analysis. The philosophy of homemade feeding differs from commercial practices, with guardians providing homemade cooked diets possibly holding favourable views towards RMBD (3). This may have led to more similar responses between the two diet groups, potentially concealing factors that predict raw feeding. Furthermore, in order for a category under a question to be included in the model, a threshold of more than 10 observations was placed. Due to the utilization of incomplete surveys and variations in selection between the two diet groups, there were instances where the predetermined threshold was not met. Hence, categories with 10 or less observations were either merged with similar options under the question to meet the threshold or omitted from analysis since they were infrequently selected. Lastly, for questions allowing participants to select or mention multiple options (e.g., multi-select questions and short-answer responses), responses were analyzed by comparing the ratio of participants who either selected/mentioned or did not select/mention the specific options between the diet groups.

## 3 Results

A total of 716 surveys were received, of which 273 did not meet the inclusion criteria and were excluded, leaving 443 surveys available for analysis. Of the 443 surveys, 74 contained complete responses to all 46 questions reported in this study.

### 3.1 Diet groups

More than half of the participants opted for commercial cooked diets (57.8%, 256/443), followed by mixed raw diets (21.0%, 93/443). The remaining participants chose commercial raw (10.8%, 48/443), mixed cooked (7.9%, 35/443), or homemade cooked diets (2.3%, 10/443), and only one participant provided an exclusively homemade raw diet. With homemade cooked feeders excluded, 67.2% (291/433) adhered to cooked feeding regimens, while 32.8% (142/433) fed partial or complete raw diets.

### 3.2 Guardian demographics

Across participants from both groups that responded to the demographic questions, most identified as female (95.3%, 243/255), residing in Canada (99.2%, 258/260), and aged between 25 and 44 years (60.1%, 152/253). Two participants in the cooked group mentioned residing in the United States and a minority from both groups identified as male (3.1%, 8/255) or non-binary/third gender (1.6%, 4/255). Additionally, many guardians had undergraduate education (60.5%, 156/258), an annual household income exceeding CA $100,000 (35.7%, 76/213), and no current or past employment in the pet care industry (75.8%, 197/260). Univariate logistic regression analyses of guardian demographics (Table 1) revealed that age and employment in pet care industry were not factors that could predict membership in a diet group. However, households with an annual income over CA $100,000 were predictive of raw feeding (*p* = 0.046, OR = 2.96), while possession of graduate education was protective against it (*p* = 0.036, OR = 0.45). Regarding the participants’ education levels, apprenticeship training or following a career in trades (2.3%, 6/258) was rarely selected and was therefore not considered in the univariate analysis.

**Table 1:** Univariate logistic regression analyses examining demographic characteristics of dog guardians feeding raw versus cooked diets.

Variable	Raw		Cooked		OR (95% CI)^*^	*p*-value^†^
	*n*	(%)	*n*	(%)		
Age (*n* = 253)						
18–24	6	(7.1)	16	(9.5)	0.92 (0.32–2.59)	0.870
25–34	27	(31.8)	66	(39.3)	Ref	
35–44	26	(30.6)	33	(19.6)	1.93 (0.97–3.81)	0.059
45–54	14	(16.5)	23	(13.7)	1.49 (0.67–3.32)	0.331
≥ 55	12	(14.1)	30	(17.9)	0.98 (0.44–2.19)	0.956
Employment in pet care industry (*n* = 260)						
Yes	21	(24.1)	42	(24.3)	0.99 (0.54–1.81)	0.980
No	66	(75.9)	131	(75.7)	Ref	
Education (*n* = 252)						
High school	4	(4.8)	12	(7.1)	0.55 (0.17–1.78)	0.317
Undergraduate	59	(71.1)	97	(57.4)	Ref	
Graduate	11	(13.3)	40	(23.7)	0.45 (0.22–0.95)	0.036
Professional degrees	9	(10.8)	20	(11.8)	0.74 (0.32–1.73)	0.487
Household income (*n* = 213)						
Less than CA $40,000	9	(12.3)	13	(9.3)	3.32 (0.92–12.01)	0.067
CA $40,000–$59,000	5	(6.9)	24	(17.1)	Ref	
CA $60,000–$79,000	13	(17.8)	23	(16.4)	2.71 (0.83–8.82)	0.097
CA $80,000–$100,000	17	(23.3)	33	(23.6)	2.47 (0.80–7.63)	0.115
Greater than CA $100,000	29	(39.7)	47	(33.6)	2.96 (1.02–8.63)	0.046

^*^Confidence intervals (CI) and Odds ratios (OR) refer to the probability of being a member of the raw group; ^†^Significance of difference compared with reference (Ref) category.

### 3.3 Dog characteristics

When assessing the dog characteristics of this cohort (Table 2), most were neutered (81.8%, 354/433) mature adults (45.9%, 196/427), with a similar sex distribution (male: 54.3%, 235/433; female: 45.7%, 198/433), and over half acquired from breeders (55.4%, 224/404). With respect to their sexual status, a higher percentage of raw feeders (23.9%, 34/142) opted to keep their dogs intact compared to cooked feeders (15.5%, 45/291). Additionally, many of the dogs were crossbreeds (43.5%, 187/430), with the remaining being purebreds. Among the purebreds, medium-sized dog breeds were more common in raw feeders (41.2%, 35/85), while large-breed dogs were most prevalent in the cooked group (39.9%, 63/158). Regarding lifestyle and environmental characteristics (Supplementary Table S1), most dogs were housed exclusively indoors (67.8%, 293/432), resided in multi-pet households (55.7%, 241/433), with most of those households having a cat present (57.9%, 139/240). Many dogs were active for either 1 hour (44.8%, 194/433) or over 2 hours (46.2%, 200/433) per day, and resided with their guardians for more than a year (82.4%, 352/427). From the above variables, possession of intact (*p* = 0.033, OR = 1.72), medium-sized (*p* = 0.017, OR = 2.85) dogs was associated with raw feeding practices. It is important to note that none of the dogs were housed primarily or exclusively outdoors, only two dogs were entirely inactive, and very few were previously strays (0.5%, 2/404) or acquired from a pet store (0.2%, 1/404). Due to the limited number of observations under these categories, they were not included in their respective univariate analysis.

**Table 2:** Univariate logistic regression analyses evaluating characteristics among dogs fed raw or cooked diets.

Variable	Raw		Cooked		OR (95% CI)^*^	*p*-value^†^
	*n*	(%)	*n*	(%)		
Age group (*n* = 427)						
Puppy	18	(12.9)	38	(13.2)	Ref	
Young adult	24	(17.3)	51	(17.7)	0.99 (0.47–2.09)	0.986
Mature adult	65	(46.8)	131	(45.5)	1.05 (0.56–1.98)	0.886
Senior	32	(23.0)	68	(23.6)	0.99 (0.49–2.00)	0.985
Sex (*n* = 433)						
Male	77	(54.2)	158	(54.3)	Ref	
Female	65	(45.8)	133	(45.7)	1.00 (0.67–1.50)	0.989
Sexual status (*n* = 433)						
Neutered	108	(76.1)	246	(84.5)	Ref	
Intact	34	(23.9)	45	(15.5)	1.72 (1.04–2.84)	0.033
Acquisition source (*n* = 401)						
Some was rehoming	17	(12.7)	28	(10.5)	Ref	
Breeder	77	(57.5)	147	(55.1)	0.86 (0.45–1.67)	0.662
Rescue	31	(23.1)	74	(27.7)	0.69 (0.33–1.44)	0.322
Friend/family member	9	(6.7)	18	(6.7)	0.82 (0.30–2.24)	0.704
Breed type (*n* = 430)						
Mixed	55	(39.3)	132	(45.5)	Ref	
Purebred	85	(60.7)	158	(54.5)	1.29 (0.86–1.95)	0.222
Breed size (*n* = 243)						
Small	9	(10.6)	33	(20.9)	Ref	
Medium	35	(41.2)	45	(28.5)	2.85 (1.21–6.74)	0.017
Large	32	(37.6)	63	(39.9)	1.86 (0.80–4.36)	0.152
Giant	9	(10.6)	17	(10.8)	1.94 (0.65–5.80)	0.235

*Confidence intervals (CI) and Odds ratios (OR) refer to the probability of being a member of the raw group; ^†^Significance of difference compared with reference (Ref) category.

### 3.4 Canine diet characteristics

In terms of diet characteristics (Supplementary Table S2), most cooked (63.2%, 177/280) and raw-fed (71.7%, 91/127) dogs had been consuming their diets for over a year. Pet food purchasing locations differed between the diet groups, with raw feeders being more likely to buy foods from pet specialty stores (*p* < 0.001, OR = 2.85) or make their own from raw ingredients (*p* < 0.001, OR = 5.99), rather than purchasing from supermarkets (*p* = 0.050, OR = 0.29) or veterinary clinics (*p* = 0.001, OR = 0.36). Although online and discount/mass retailers were also selected, purchasing pet food from these locations did not predict membership in a diet group. Hardware stores were mentioned by few participants (0.7%, 3/432) as a source for purchasing pet food, therefore a univariate analysis was not conducted. Regarding the components in diets of dogs fed human food ingredients, among cooked feeders, the top five ingredients were vegetables (62.1%, 18/29), fruits (48.3%, 14/29), red meat (41.4%, 12/29), grains (37.9%, 11/29), and poultry (31.0%, 9/29). For the raw group, vegetables (57.6%, 19/33), red meat (36.4%, 12/33), poultry (27.3%, 9/33), organs (24.2%, 8/33), fruits (24.2%, 8/33), and eggs (24.2%, 8/33) were common ingredient choices. Given that homemade cooked feeders were excluded and only one homemade raw feeder was identified in this cohort, these ingredients reflect the choices of guardians predominantly adhering to mixed feeding practices. Moreover, most participants (70.3%, 296/421) reported no changes to their pet’s diet in the past 4–6 months. Of those who did make changes, 1 of 87 cooked feeders (1.1%) reported previously providing raw elements. During the same period, 21.1% (8/38) of raw feeders introduced raw food and 5.3% (2/38) integrated cooked ingredients. Regarding the guardians’ plans to change their pets’ diets in the next 4–6 months, most participants (68.7%, 288/419) reported no intention to make any adjustments. Among those considering a dietary change, a small percentage of cooked feeders (9.7%, 3/31) expressed a desire to transition to partial or full raw diets, while none of the raw feeders reported an interest in switching to a fully cooked diet.

Supplement provision varied between the diet groups, with more raw feeders (67.7%, 84/124) including supplements in their dogs’ diets compared to cooked feeders (39.0%, 105/269). The most commonly mentioned supplements were joint supplements (36.2%, 63/174), fish oil (29.3%, 51/174), probiotics (23.6%, 41/174), and multi-vitamin/mineral mixes (8.6%, 15/174). However, supplementation with fish oil was more popular among raw-fed dogs (40.8%, 31/76) compared to cooked-fed dogs (20.4%, 20/98). When asked about the reasons for providing supplements, many respondents mentioned to improve pet health (56.9%, 107/188), because it was recommended by veterinarians who were not board-certified nutritionists (39.9%, 75/188), or to ensure the diet was complete and balanced (37.8%, 71/188). Less popular recommendation sources included books or online sources (13.8%, 26/188), board-certified veterinary nutritionists (13.3%, 25/188), breeders (12.8%, 24/188), friends or family members (10.1%, 19/188), veterinary technicians/nurses (10.1%, 19/188), pet or retail stores (7.4%, 14/188), pet food manufacturers (6.9%, 13/188), and shelters (0.5%, 1/188), with none of the respondents providing supplements based on recommendations from the pet’s previous guardian. Univariate analyses indicated the provision of supplements (*p* < 0.001, OR = 3.28) to ensure diet completeness and balance (*p* < 0.001, OR = 4.59) were predictive of raw feeding, with the addition of fish oil (*p* = 0.004, OR = 2.69) raising the odds of being in the raw group. In contrast, the provision of supplements based on the recommendations of veterinarians who were not specialized in nutrition (*p* = 0.491, OR = 0.02) was protective against raw feeding. Lastly, providing supplements based on the recommendations of pet food manufacturers was also predictive of raw feeding (*p* = 0.006, OR = 18.00), but only one participant who fed cooked diets selected this option, resulting in a prediction with a wide confidence interval.

### 3.5 Guardians’ dog food choices and knowledge of dog nutrition

Knowledge perceptions about canine nutrition varied among the diet groups (Supplementary Table S3). Perceiving oneself as moderately (*p* = 0.032, OR = 2.13) or very (*p* = 0.007, OR = 2.76) knowledgeable in canine nutrition increased the likelihood of feeding raw diets. Guardians who also thought about their dog’s nutrition with every meal were more likely to feed raw (*p* = 0.011, OR = 2.51). Considering only one participant (0.2%, 1/427) reported never thinking about their dog’s nutrition, the category was excluded from the univariate model. Regarding the guardians’ level of satisfaction with their dog’s current diet, most raw (69.1%, 85/123) and cooked (61.1%, 165/270) feeders reported extreme satisfaction. When questioned on how processed, preserved, or raw they considered their dog’s diets to be, perceiving the diets as minimally processed (*p* < 0.001, OR = 6.56), marginally preserved (*p* < 0.001, OR = 3.88), and more raw (*p* < 0.001, OR = 8.26) was predictive of raw feeding. Conversely, perceiving them as very processed (*p* = 0.027, OR = 0.53) and minimally raw (*p* < 0.001, OR = 0.22) decreased the likelihood of being a raw feeder.

In addition to the above differences, factors affecting pet food selection also varied between cooked and raw feeders (Supplementary Table S4). The odds of being in the raw group significantly increased if the following factors/claims influenced pet food selection: environmental friendliness/sustainability (*p* = 0.005, OR = 2.04), freshness (*p* < 0.001, OR = 3.57), grain-free (*p* < 0.001, OR = 2.84), non-GMO (*p* < 0.001, OR = 3.37), homemade (*p* = 0.010, OR = 2.96), organic (*p* = 0.003, OR = 3.10), natural/holistic (*p* < 0.001, OR = 5.50), similar to an ancestral diet (*p* < 0.001, OR = 7.97), minimal processing (*p* < 0.001, OR = 3.87), no artificial additives or preservatives (*p* < 0.001, OR = 3.23), high quality ingredients (*p* < 0.001, OR = 2.87), meat first as an ingredient (*p* < 0.001, OR = 2.41), presence of raw ingredients (*p* < 0.001, OR = 50.60), and if it was recommended by friends/family members (*p* = 0.042, OR = 2.16). In contrast, the odds of feeding raw significantly decreased if the subsequent factors influenced pet food selection: feeding convenience (*p* = 0.011, OR = 0.55), price value (*p* = 0.016, OR = 0.55), and if it was recommended by board-certified veterinary nutritionists (*p* = 0.012, OR = 0.55), veterinarians who were not specialized in nutrition (*p* < 0.001, OR = 0.22), or veterinary technicians/nurses (*p* = 0.003, OR = 0.32). Factors that could not predict diet group membership (*p* > 0.05) were: benefits to skin and coat health, nutritional completeness and balance, purchasing convenience, digestibility, easy access package/storage capability, inclusion and/or exclusion of ingredients, fecal consistency, flavour/palatability, internet reviews, pet preference, prevention and/or treatment of ailment/condition, reputation of the company, feeding safety, if the product included grains or gluten, or if they were recommended by breeders, shelters, previous guardians, and retail stores. The influence of manufacturers’ recommendations when selecting pet food was also listed, but with only 2.0% (8/394) of guardians selecting this option, a univariate analysis was not conducted.

Regarding the nine statements surrounding beliefs and considerations of dog guardians in canine nutrition (Supplementary Table S5), differences in viewpoints emerged between the diet groups. Agreement with the statement outlining the importance of pet food labels and ingredient lists when selecting pet food was associated with raw feeding (*p* = 0.017, OR = 3.07), as was the belief that homemade (*p* = 0.027, OR = 2.07) and raw meat-based diets (*p* < 0.001, OR = 5.44) are nutritionally superior to heat-processed commercial products. The belief that dogs are carnivores and require meat-based diets (*p* < 0.001, OR = 3.00) along with variety in day-to-day meals (*p* < 0.001, OR = 3.62) also increased the likelihood of feeding raw. However, believing that heat-processed commercial pet food contains all the nutrients a pet needs decreased the odds of being a raw feeder (*p* < 0.001, OR = 0.20). Additionally, the likelihood of observing raw feeding practices decreased if guardians disagreed with the statements surrounding the nutritional superiority of homemade (*p* < 0.001, OR = 0.24) and RMBD (*p* = 0.002, OR = 0.24) over heat-processed commercial pet food. The guardians’ level of agreement with the statements about grains being nutritious or cooking degrading nutrients did not predict membership in any diet group. Regardless, it is worth noting that 40.3% (29/72) of raw feeders disagreed that grains are good nutrient sources for pets whereas 39.5% (60/152) of cooked feeders were neutral on this statement. For the statement outlining the risk of nutrient destruction with cooking, raw feeders (42.2%, 35/83) were generally neutral, in contrast to cooked feeders (47.0%, 63/134) who tended to disagree. Lastly, most guardians across both groups agreed that nutrition is important for dog health (96.9%, 252/260). For the latter, considering only a small percentage of guardians across both groups were neutral (2.3%, 6/260) or disagreed (0.8%, 2/260) with this statement, a univariate analysis was not conducted. Similarly, in the univariate analysis regarding the importance of pet food labels and ingredient lists for pet food selection, participants who disagreed (1.9%, 5/259) were excluded due to the limited number of observations under this category.

### 3.6 Guardians’ nutrition choices and knowledge of human nutrition

Survey questions surrounding the guardians’ own nutrition choices and knowledge of human nutrition (Supplementary Table S6) were unable to predict membership in a diet group. Raw feeders (46.7%, 43/92) frequently reported thinking about their personal nutrition regularly but not daily, whereas cooked feeders (45.3%, 82/181) often thought about their nutrition with every meal. Additionally, only 2.2% (6/273) of respondents stated they never thought about their own nutrition, but these participants were excluded in the univariate analysis due to the limited number of observations. In terms of perceived diet health, cooked (46.4%, 83/179) and raw feeders (57.6%, 53/92) frequently considered their diet to be moderately healthy. Regarding dietary preferences, most participants were omnivores (88.1%, 237/269), with only a few following non-omnivorous diets, including vegetarian (4.5%, 12/269), pescatarian (4.1%, 11/269), carnivorous (1.9%, 5/269), or vegan (1.5%, 4/269) diets. Furthermore, a minority of guardians (19.0%, 52/273) recognized adhering to dietary trends or restrictions. The most common in the cooked group was a low dairy or dairy-free diet (18.4%, 7/38), while raw feeders mentioned a diet containing low or no gluten (42.9%, 6/14). Most guardians (86.0%, 234/272) also reported being the primary meal preparers in their household, allocating between 30 min to an hour each day on this task (49.6%, 135/272). Meal preparation or batch cooking was not widely practiced within this cohort (20.0%, 54/270); however, among those who stated engaging in such activity, this was done 2 days per week or less (68.5%, 37/54). Lastly participants were asked about their perceived level of knowledge about human nutrition and their confidence in making self-directed dietary changes. Across both groups, participants often expressed moderate knowledgeable (60.5%, 159/263) and confidence (49.4%, 130/263) about making dietary changes. Two participants also reported no knowledge of human nutrition, but they were not considered in the univariate analysis due to the limited number of observations.

### 3.7 Human-animal bond

Similarly to the previous section, survey questions assessing human-animal relationships (Supplementary Table S7) could not predict diet group membership. Both groups primarily considered their dogs as companions (94.5%, 409/433), with only a few participants viewing them as sports/agility (9.2%, 40/433), working (5.1%, 22/433), obedience (5.1%, 22/433), breeding (4.2%, 18/433), or showing (3.0%, 13/433) pets. Hunting was also an option, but only 1.2% (5/433) selected this category, therefore a univariate analysis was not conducted. In terms of how the participants perceived their dog-guardian relationship, the majority across both groups viewed themselves as either pet parents (42.2%, 181/429) or perceived their relationship as akin to that of family members (32.6%, 140/429). Few guardians considered themselves as owners (14.5%, 62/429) or regarded the relationship solely for companionship purposes (8.6%, 37/429). Less common perceptions, such as friend (1.6%, 7/429) and caregiver (0.5%, 2/429), were not included in the univariate model.

## 4 Discussion

Compared to guardians who fed cooked pet food, those who chose raw diets showcased differences in demographics, dog characteristics and diets, pet food choices and perceived knowledge of dog nutrition, as well as opinions about canine diets; however, questions regarding guardians’ nutrition and viewpoints of their human-animal relationships could not predict membership in a diet group.

While it is challenging to determine the exact prevalence of pet feeding practices, the diet distribution in this sample aligns with previous surveys of dog guardians. Cooked pet food diets, primarily in the form of commercial kibble, are often reported as the preferred choice for most guardians (21–23). In comparison, partial or exclusive raw diets seem to be less common, which accounts for the disproportionate diet groups in this study (21–23). Although exclusive raw feeding may be less frequent than partial raw feeding (2), it was surprising that only one participant fed an exclusive homemade raw diet. This suggests that raw feeders within this sample primarily reflect characteristics and perspectives of guardians adhering to commercial or mixed raw feeding practices. Future research should recognize that guardians who report feeding RMBD may primarily provide partial raw diets, and account for this distinction when interpreting results.

In this study, almost all participants were Canadian. Currently, there is limited research voicing the views of Canadian dog guardians on raw diets. However, since many participants did not mention their place of origin, it is possible that the non-respondents were also Canadian, or that a larger portion of the participants resided in the United States or other countries. Moreover, as with this sample, pet guardian survey studies mostly represented females in their early to mid-adulthood (4, 5, 11, 23–25). Male dog guardians were underrepresented, meaning gender-related differences in raw feeding practices remain unclear and may have affected survey findings. Regarding participant income, research has shown that it can influence both pet guardianship and pet food purchasing habits, as the cost of pet food has been cited as a factor influencing adherence to raw feeding practices (14–16, 26). In this study, higher income was associated with raw feeding, while prioritizing price value when selecting pet food decreased the likelihood of adopting raw diets. Thus, guardians who choose raw diets could be more affluent, which allows them to support these niche diets that may be more expensive than conventional options. In addition, previous studies indicate that most guardians have at minimum a high school diploma and often pursue higher education, with those holding tertiary education being more likely to inspect label ingredients and claims of pet food products (1, 13, 15, 17, 23, 26, 27). Findings from the current study suggest these qualities may be more commonly associated with raw feeders, implying that guardians who practice raw feeding may generally be more well-educated. However, graduate education was found to be a protective factor against raw feeding. A similar trend was noted among guardians with doctorate degrees, who were less likely to choose commercial raw diets, whereas guardians with some college education tended to feed RMBD more often (28). Therefore, the extent to which education may predict raw feeding practices might depend on the specific field of study, as this could influence perspectives on raw diets. With regards to guardian age, age groups 25–34 and 45–54 may be associated with raw feeding practices (28). Interestingly, guardians under 35 may place more importance on cruelty-free and grain-free claims than those over 65 (13). While these traits could be linked to raw feeders, age alone did not predict raw feeding in this study. The sample’s age distribution, which mainly consisted of individuals between 25 and 44, may have prevented such prediction due to the concentration of participants within a narrower age range.

Research on the relationship between dog characteristics and raw feeding practices remains limited. A survey study on Italian dog guardians who feed RMBD found most dogs to be mature adults, which aligns with the results of the present study (4). In addition, although prior research suggests senior dogs may be less likely to receive raw diets, dog age did not predict raw feeding practices in this sample as age distributions were similar between cooked and raw-fed dogs (6, 29). With respect to breed size, medium and large breeds may be associated with raw feeding practices (4, 6). While the present study found a significant link only with medium-sized dogs, raw diets may be more frequently selected for larger breeds due to the belief that protein-rich, meat-dominant diets better meet the nutritional needs of such dogs (4). Furthermore, although most of the dogs in this cohort were neutered, intact dogs are more likely to consume raw diets according to this study and past research (4, 6, 28). Given the raw feeders in this group were compelled to honour their dogs’ ancestral meat-based eating habits, they might also prefer to keep their dogs intact in order to respect their natural reproductive state. While raw feeders have been reported to live in multi-pet households, this study did not observe such relationship (6). Considering cats’ carnivorous nature, a univariate analysis examined whether feline companions influenced the provision of raw canine diets; however, no association was found, leaving the impact of other pets on RMBD adoption unclear. Furthermore, neither dog breed type nor acquisition source was found to influence raw feeding in this cohort. In line with the findings of this study, breeders and shelters are more frequently noted as the main sources for acquiring pets (27, 29–31). While a univariate analysis of the dogs’ acquisition sources could not predict diet group membership, obtaining diet information from pet breeders has been linked to raw feeding practices (6). A 2024 study also suggested that purebreds may be more likely to consume raw diets, potentially reflecting the role of breeders in shaping guardians’ feeding decisions (28). Therefore, even if the acquisition of pets from breeders may not be directly related to raw feeding, breeders could still influence the adoption of RMBD through their nutritional guidance. Dog outdoor access was also examined but did not influence raw diet provision, possibly because most participants were Canadian, where colder climates may have kept many dogs indoors. However, this survey did not assess the influence of guardian activity or urban versus rural residential setting on raw feeding; thus, future studies should explore how these variables influence the adoption of RMBD.

The assessment of canine diets revealed differences between cooked and raw feeding practices. Guardians feeding raw diets favoured purchasing dog food from pet specialty stores rather than supermarkets, potentially due to the greater availability of RMBD in these stores. The reduced likelihood of buying RMBD from veterinary clinics may reflect veterinarians’ reluctance to endorse raw diets due to existing concerns about nutritional imbalances and microbial risks (4, 5, 7–10, 32–34). Considering only one guardian fed an exclusive homemade raw diet, the link between purchasing raw ingredients for dog food preparation and the raw feeders of this sample may mirror mixed feeding or a desire for greater control over their dogs’ diets. In terms of ingredients, raw feeders used more animal-based components, whereas cooked feeders incorporated a greater proportion of fruits and grains. The notable presence of vegetables in the raw-fed group could also be indicative of mixed raw feeding practices, since raw feeders may prefer to provide diets with fewer carbohydrates (4). In this study, offering supplements corresponded with a greater probability to feed raw. The increased supplementation could suggest raw feeders have concerns about the nutrient content of these diets or perceive benefits in offering dietary supplements. Moreover, fish oil inclusion was associated with raw feeding practices, supporting previous research that observed frequent addition of marine-derived omega-3 supplements in RMBD (4, 35). This may explain why raw-fed dogs are often reported to have shinier coats, as the higher dietary fat content and fish oil supplementation in these diets could contribute to improved skin and coat health (4, 6, 10, 36). However, it should be noted that the low response rate to follow-up questions on supplement use may have resulted in underreporting of certain practices. Considering raw feeders’ distrust of veterinarians, it is unsurprising that raw feeding was less likely when supplement provision was influenced by non-board-certified veterinary nutritionists, underscoring the limited role of veterinarians in shaping these guardians’ feeding choices (4–6, 17). While recommendations from pet food companies appeared to impact supplement provision, the wide confidence interval for this estimate stemmed from the small number of observations in this category. Low sample size may affect estimate stability and suggest that, despite statistical significance, it may not reliably predict raw feeding in this sample of dog guardians.

Differences between the diet groups were evident regarding dog food choices and perceived knowledge of dog nutrition. Raw feeders both in this sample and prior research viewed themselves as more knowledgeable about dog nutrition than cooked diet feeders (5, 15). Given that raw feeding practices are motivated by delivering unprocessed and natural ingredients, it was not surprising that raw feeders of this sample were more likely to view their dogs’ diets as less preserved, minimally processed, and more raw (4, 6, 17). Specifically, guardians recognize natural ingredients as a marker of pet food quality, with raw feeders in this sample also considering ingredient processing when selecting diets (5, 6, 11–13). However, choices were further shaped by specific ingredients (e.g., grain-free), the order of ingredients in labels (e.g., meat listed first), and recommendations from friends or family. Although high cost and time demands may deter some guardians from feeding RMBD, our sample indicates that raw feeders are less influenced by these factors, suggesting that the raw-feeding guardians in this cohort could manage the associated demands (14). Similar to supplement provision, the influence of veterinary professionals on diet selection reduced the likelihood of raw feeding. This may trace back to raw feeders’ distrust of veterinarians, as previous studies have shown that they often turn to unverified sources for feeding guidance, viewing veterinarians as less credible for nutritional advice (4–6, 17). Perceived benefits of raw feeding, such as improved skin, coat, fecal consistency, and digestibility did not influence diet selection among raw feeders. This suggests that ingredient lists and product claims, rather than health benefits, could primarily drive RMBD selection. Alternatively, respondent fatigue may have contributed to the non-significant results, given that participants had 41 factors to evaluate. Regarding the statements exploring beliefs and considerations about canine nutrition, raw feeders viewed homemade and raw diets as nutritionally superior to commercial kibble and wet pet foods, potentially due to the perception that commercial cooked diets conflict with dogs’ ancestral carnivorous consumption habits. Similar views were held among guardians who fed non-commercial diets, such as homemade meals and human foods (3); however, unlike the raw feeders in this sample, the non-commercial feeders considered cooking to reduce nutrients in pet food. Furthermore, grains were seen as poor nutrient sources by the non-commercial feeders (3); yet, while most raw feeders in this sample agreed with such viewpoint, the univariate analysis did not predict diet group membership. Variations in opinions may stem from differences in diet composition, as most raw feeders in this study followed mixed feeding practices, which could have included commercial and homemade cooked elements to varying degrees; hence, future studies should investigate potential differences in opinions or traits between raw feeding subgroups. It is important to also acknowledge that, in statements where raw feeders of this sample expressed more favourable views towards homemade diets, the exclusions of homemade cooked feeders in this study may have amplified differences between the diet groups. Considering homemade cooked feeders would likely have favoured their own feeding type, such inclusion may have led to responses from the cooked group becoming more similar to those of raw feeders, potentially lowering the likelihood of detecting significant associations. Responses to survey questions not related to homemade cooked feeding practices may also have differed if homemade cooked feeders had been included, although the direction and magnitude of such differences cannot be determined from the present data. Given that raw feeders may hold favourable views towards homemade diets regardless of preparation method, future research could examine the alignment of opinions and feeding practices between the two groups to better understand non-commercial feeding methods among dog guardians.

Survey questions exploring participants’ own dietary habits could not predict membership in a diet group. A study reported that first-year veterinary students in Canada and the United States generally rated their diet as moderately healthy; however, students with higher levels of physical activity were more likely to have a positive perception of their diet’s healthfulness (37). Therefore, variations in the participants’ lifestyles may have influenced the effectiveness of human nutrition variables in predicting diet group membership. Since most of the participants in this survey were female, gender-related variations in dietary patterns may have also prevented such predictions (38, 39). Regarding the frequency with which participants thought about their personal nutrition, this variable did not predict diet group membership; however, thinking about a dog’s nutrition with each meal was linked to raw feeding practices. Similarly, those who viewed themselves as more knowledgeable about dog nutrition were more likely to choose raw diets, while their perceived understanding of human nutrition had no influence on their group membership. Although some survey questions were significant in the context of dog nutrition, none of the responses related to the guardians’ own nutrition predicted raw feeding. This suggests that the provision of raw diets may be driven more by the remaining factors explored in this study rather than the guardians’ personal dietary choices. However, it is crucial to also question whether a more balanced ratio of male to female respondents or understanding participants’ lifestyles and level of physical activity could have shed light on the role of these factors in adopting raw feeding methods. Future studies could also examine the frequency of raw vegetable or meat consumption among participants, as these habits may influence guardians’ viewpoints on raw feeding practices.

As with human consumption practices, participants’ perception of their human-animal relationships did not influence diet group membership. Previous studies have highlighted that most guardians consider their pets as family members, with companionship being the main purpose of the relationship (21, 27, 31). Additionally, viewing pets as family members may lead individuals to prioritize their pet’s diet over their personal nutrition, which may also make them less sensitive to the cost of dog food compared to their own food expenses (16, 40). Therefore, as the bond between pets and guardians strengthens, pet food selection practices may shift to prioritize nutritional quality and overall eating experience (1). For example, guardians who choose natural, unprocessed foods for themselves may also be more inclined to apply the same health-centric habits to their pets (16, 17). Given the raw feeders in this group appeared more concerned with nutritional quality and ingredients, it was anticipated that their preference for raw diets was influenced by their own healthier eating habits and a stronger bond with their dogs. However, the present study did not find such an association, as most guardians across diet groups reported consuming an omnivorous diet and viewed their pets as companions or themselves as pet parents. Hence, it is important to consider if positive views on human-animal relationships are associated with dog guardianship in general and not necessarily translate into a preference for raw feeding practices. Interestingly, working dogs may be more likely to be fed homemade raw diets, while service dogs could be linked to commercial raw feeding methods (28). As a result, dogs that are expected to perform energy-demanding tasks could be associated with RMBD, as these diets may be perceived as healthier or better suited for their needs. Given that most participants viewed their dogs as companions and very few perceived them as working animals, this may also explain why this factor was not linked to raw feeders in this study. Future research should explore this topic more thoroughly among raw feeders by examining how dogs affect personal activities, daily routines, and social relationships to better understand the connection between canine diet selection and the perceived role of pets in guardians’ lives.

Regarding the study’s limitations, all respondents required internet access and proficiency in English to participate in the study, which may have resulted in the exclusion of guardians who lacked these capabilities. Selection bias may have also occurred due to the voluntary nature of participation, likely attracting individuals with a stronger-than-average interest in dog nutrition, thereby limiting the generalizability of the findings to the wider population of dog guardians. Furthermore, the imbalance in the number of cooked and raw feeders in this sample may have masked certain differences in feeding practices between the diet groups. It is crucial to also account for the potential impact of non-response bias, as the number of observations to some survey questions was occasionally lower than the total sample size. Considering the high number of questions in the survey instrument, respondents likely devoted greater attention to the initial questions, while later questions may have received less consideration or led respondents to exit the survey. If a greater number of complete surveys had been collected, the findings could have shifted, or additional predictive categories for raw feeding practices might have emerged. Lastly, the reliance on multiple univariate logistic regression analyses to draw conclusions may have increased the risk of false-positive associations.

## 5 Conclusion

Exploratory survey data from 433 dog guardians suggest that those choosing RMBD tend to differ from guardians feeding cooked pet food. Demographic variables, including income and education, appeared to vary between individuals who feed raw components and guardians choosing cooked diets. The selection of RMBD could also be influenced by dog characteristics, such as breed size and sexual status. Moreover, diets of raw-fed dogs showed variations from those consuming cooked foods in terms of where the commercial diets were purchased and the use of dietary supplements. Variations may also exist in raw feeders’ perceptions of canine nutrition knowledge, dog food selection, and attitudes towards feeding practices. However, differences between raw and cooked feeders may not be evident based on human nutrition choices and perceptions of human-animal relationships. These findings could help pet food manufacturers optimize product formulation and marketing strategies to more effectively reach audiences exhibiting characteristics similar to raw feeders. Likewise, veterinary professionals can use this information to better understand raw-feeding practices, enabling more personalized nutritional recommendations and potentially increasing guardian compliance with veterinary advice. Subsequent studies could examine the manner in which pet-guardian relationships and human nutrition habits shape raw feeding practices, particularly among partial versus exclusive raw feeders.

## Data Availability

The raw data supporting the conclusions of this article will be made available by the authors, without undue reservation.

## References

[1] Boya UO Dotson MJ Hyatt EM. A comparison of dog food choice criteria across dog owner segments: an exploratory study. Int J Consum Stud. (2015) 39:74–82. doi: 10.1111/ijcs.12145

[2] Dodd S Cave N Abood S Shoveller A-K Adolphe J Verbrugghe A. An observational study of pet feeding practices and how these have changed between 2008 and 2018. Vet Rec. (2020) 186:643–9. doi: 10.1136/vr.105828, 32554799

[3] Michel KE Willoughby KN Abood SK Fascetti AJ Fleeman LM Freeman LM . Attitudes of pet owners toward pet foods and feeding management of cats and dogs. J Am Vet Med Assoc. (2008) 233:1699–703. doi: 10.2460/javma.233.11.1699, 19046026

[4] Morelli G Bastianello S Catellani P Ricci R. Raw meat-based diets for dogs: survey of owners’ motivations, attitudes and practices. BMC Vet Res. (2019) 15:1–10. doi: 10.1186/s12917-019-1824-x, 30832667 PMC6399943

[5] Empert-Gallegos A Hill S Yam PS. Insights into dog owner perspectives on risks, benefits, and nutritional value of raw diets compared to commercial cooked diets. PeerJ. (2020) 8:e10383. doi: 10.7717/peerj.10383, 33354417 PMC7731655

[6] Morgan G Williams N Schmidt V Cookson D Symington C Pinchbeck G. A dog’s dinner: factors affecting food choice and feeding practices for UK dog owners feeding raw meat-based or conventional cooked diets. Prev Vet Med. (2022) 208:1–10. doi: 10.1016/j.prevetmed.2022.105741, 35994979

[7] Bottari B Bancalari E Barera A Ghidini S Gatti M. Evaluating the presence of human pathogens in commercially frozen, biologically appropriate raw pet food sold in Italy. Vet Rec. (2020) 187:e50–6. doi: 10.1136/vr.105893, 32430390

[8] van Bree FPJ Bokken GCAM Mineur R Franssen F Opsteegh M van der Giessen JWB . Zoonotic bacteria and parasites found in raw meat-based diets for cats and dogs. Vet Rec. (2018) 182:50–7. doi: 10.1136/vr.104535, 29326391

[9] Dodd S Barry M Grant C Verbrugghe A. Abnormal bone mineralization in a puppy fed an imbalanced raw meat homemade diet diagnosed and monitored using dual-energy x-ray absorptiometry. J Anim Physiol Anim Nutr. (2021) 105:29–36. doi: 10.1111/jpn.13118, 31144390

[10] Vecchiato CG Schwaiger K Biagi G Dobenecker B. From nutritional adequacy to hygiene quality: a detailed assessment of commercial raw pet-food for dogs and cats. Animals. (2022) 12:1–17. doi: 10.3390/ani12182395, 36139257 PMC9495138

[11] Prata JC. Survey of pet owner attitudes on diet choices and feeding practices for their pets in Portugal. Animals. (2022) 12:1–14. doi: 10.3390/ani12202775, 36290160 PMC9597766

[12] Rajagopaul S Parr JM Woods JP Pearl DL Coe JB Verbrugghe A. Owners’ attitudes and practices regarding nutrition of dogs diagnosed with cancer presenting at a referral oncology service in Ontario, Canada. J Small Anim Pract. (2016) 57:484–90. doi: 10.1111/jsap.12526 27357412

[13] Vinassa M Vergnano D Valle E Giribaldi M Nery J Prola L . Profiling Italian cat and dog owners’ perceptions of pet food quality traits. BMC Vet Res. (2020) 16:131–10. doi: 10.1186/s12917-020-02357-9, 32393389 PMC7216655

[14] Baum LL Zablotski Y Busch K Koelle P. Reasons why dog owners stop feeding raw meat-based diets (RMBDs) - an online survey. Pets. (2024) 1:20–32. doi: 10.3390/pets1010004

[15] Rombach M Dean DL. It keeps the good boy healthy from nose to tail: understanding pet food attribute preferences of US consumers. Animals. (2021) 11:1–15. doi: 10.3390/ani11113301, 34828032 PMC8614497

[16] Tesfom G Birch N. Do they buy for their dogs the way they buy for themselves? Psychol Mark. (2010) 27:898–912. doi: 10.1002/mar.20364

[17] Morgan SK Willis S Shepherd ML. Survey of owner motivations and veterinary input of owners feeding diets containing raw animal products. PeerJ. (2017) 5:1–16. doi: 10.7717/peerj.3031, 28265510 PMC5337082

[18] Harvey ND. How old is my dog? Identification of rational age groupings in pet dogs based upon normative age-linked processes. Front Vet Sci. (2021) 8:1–6. doi: 10.3389/fvets.2021.643085, 33987218 PMC8110720

[19] Canadian Kennel Club. Choosing a breed. (2014) Available online at: https://www.ckc.ca/en/Choosing-a-Dog/Choosing-a-Breed (Accessed July 11, 2024).

[20] Salt C Morris PJ German AJ Wilson D Lund EM Cole TJ . Growth standard charts for monitoring bodyweight in dogs of different sizes. PLoS One. (2017) 12:e0182064. doi: 10.1371/journal.pone.0182064, 28873413 PMC5584974

[21] Joosten P Cleven AV Sarrazin S Paepe D Sutter AD Dewulf J. Dogs and their owners have frequent and intensive contact. Int J Environ Res Public Health. (2020) 17:1–10. doi: 10.3390/ijerph17124300, 32560155 PMC7345801

[22] Laflamme DP Abood SK Fascetti AJ Fleeman LM Freeman LM Michel KE . Pet feeding practices of dog and cat owners in the United States and Australia. J Am Vet Med Assoc. (2008) 232:687–94. doi: 10.2460/javma.232.5.687, 18312173

[23] Schleicher M Cash SB Freeman LM. Determinants of pet food purchasing decisions. Can Vet J. (2019) 60:644–50. 31156266 PMC6515811

[24] Evason M Peace M Munguia G Stull J. Clients’ knowledge, attitudes, and practices related to pet nutrition and exercise at a teaching hospital. Can Vet J (2020) 61:512–516. PMCID: 32355350 PMC7155884

[25] Kamleh M Khosa DK Verbrugghe A Dewey CE Stone E. A cross-sectional study of pet owners’ attitudes and intentions towards nutritional guidance received from veterinarians. Vet Rec. (2020) 187:1–7. doi: 10.1136/vr.105604, 33272957

[26] Forrest R Awawdeh L Pearson M Waran N. Pet ownership in Aotearoa New Zealand: a national survey of cat and dog owner practices. Animals. (2023) 13:1–16. doi: 10.3390/ani13040631, 36830418 PMC9951667

[27] Howell TJ Mornement K Bennett PC. Pet dog management practices among a representative sample of owners in Victoria, Australia. J Vet Behav. (2016) 12:4–12. doi: 10.1016/j.jveb.2015.12.005

[28] O’Brien JS Tolbert MK Ruple A. Dog and owner demographics impact dietary choices in dog aging project cohort. J Am Vet Med Assoc. (2024) 262:1676–85. doi: 10.2460/javma.24.05.0358, 39142333 PMC12093940

[29] Wallis LJ Szabó D Erdélyi-Belle B Kubinyi E. Demographic change across the lifespan of pet dogs and their impact on health status. Front Vet Sci. (2018) 5:1–20. doi: 10.3389/fvets.2018.00200, 30191153 PMC6115627

[30] Dodd S Khosa D Dewey C Verbrugghe A. Owner perception of health of north American dogs fed meat- or plant-based diets. Res Vet Sci. (2022) 149:36–46. doi: 10.1016/j.rvsc.2022.06.002, 35717887

[31] Gates MC Walker J Zito S Dale A. Cross-sectional survey of pet ownership, veterinary service utilisation, and pet-related expenditures in New Zealand. N Z Vet J. (2019) 67:306–14. doi: 10.1080/00480169.2019.1645626, 31319781

[32] American Animal Hospital Association. Raw protein diet. (2021). Available online at: https://www.aaha.org/resources/2021-aaha-nutrition-and-weight-management-guidelines/raw-protein-diet/ (Accessed November 24, 2025).

[33] Canadian Veterinary Medical Association. Safety of raw meat-based pet food products. (2023). Available online at: https://www.canadianveterinarians.net/policy-and-outreach/position-statements/statements/safety-of-raw-meat-based-pet-food-products/ (Accessed November 24, 2025).

[34] World Small Animal Veterinary Association. Raw meat based Diets for pets. (2020). Available online at: https://wsava.org/wp-content/uploads/2021/04/Raw-Meat-Based-Diets-for-Pets_WSAVA-Global-Nutrition-Toolkit.pdf (Accessed November 24, 2025).

[35] Morelli G Stefanutti D Ricci R. A survey among dog and cat owners on pet food storage and preservation in the households. Animals. (2021) 11:1–19. doi: 10.3390/ani11020273, 33494534 PMC7911149

[36] Logas D Kunkle GA. Double-blinded crossover study with marine oil supplementation containing high-dose eicosapentaenoic acid for the treatment of canine pruritic skin disease. Vet Dermatol. (1994) 5:99–104. doi: 10.1111/j.1365-3164.1994.tb00020.x, 34645070

[37] Nielson SA Kamleh MK Conlon PD McWhirter JE Stone EA Khosa DK. Understanding incoming Canadian and US veterinary students’ attitudes and perceptions of their dietary habits and levels of physical activity. J Vet Med Educ. (2021) 48:747–55. doi: 10.3138/jvme-2020-0065, 33657337

[38] Boek S Bianco-Simeral S Chan K Goto K. Gender and race are significant determinants of students’ food choices on a college campus. J Nutr Educ Behav. (2012) 44:372–8. doi: 10.1016/j.jneb.2011.12.007, 22607739

[39] Li K-K Concepcion RY Lee H Cardinal BJ Ebbeck V Woekel E . An examination of sex differences in relation to the eating habits and nutrient intakes of university students. J Nutr Educ Behav. (2012) 44:246–50. doi: 10.1016/j.jneb.2010.10.002, 21764641

[40] Kamleh MK Khosa DK Dewey CE Verbrugghe A Stone EA. Ontario veterinary college first-year veterinary students’ perceptions of companion animal nutrition and their own nutrition: implications for a veterinary nutrition curriculum. J Vet Med Educ. (2021) 48:71–83. doi: 10.3138/jvme.0918-113r1, 32412363

