# Supplementary material for: Insights into the influence of dog and guardian demographics, nutrition, and relationship on raw feeding practices
Source: Front Vet Sci. 2026 May 20;13:1793754. doi: 10.3389/fvets.2026.1793754 (PMC13231891; doi:10.3389/fvets.2026.1793754)
Supplement: Supplementary file 1 [file Data_Sheet_1.PDF]

Pet Guardian Survey

Start of Block: Consent

Survey of Dog Owners' Perspectives on Different Pet Food Types: Participant Consent Form

You are invited to take part in a research project with the intended goal of gaining a better understanding of dog owners' perspectives on cooked and raw diets. A second goal is to understand what drives dog owners' decisions to select a specific diet type. You are eligible to participate in this study if you are the primary person who makes dietary decisions for a dog and are 18 years of age or older.

In order to protect confidentiality, PERSONAL (IDENTIFYING) INFORMATION will NOT be collected at any time as part of the main survey unless you wish to be entered into a draw. Identifiers for entering the draw will be collected in a separate survey, but because Qualtrics automatically assigns timestamps to survey submission, there is a small possibility in the event of a data breach that the identifiers could be linked to the survey responses via the timestamps, though the researchers will make no attempt to do so. After the draw all email addresses will be deleted.

If you decide to provide your email address to enter into a draw, you will have a 1 IN 100 CHANCE OF WINNING one of the four $30 AMAZON GIFT CARDS. The researchers will use your email address to contact you, only if you are selected as a winner. You will receive two emails within 10 days. If a response to claim the prize is not received, a replacement winner will be selected.

As the survey is anonymous, data provided by you may NOT be withdrawn from the research project once your completed survey is submitted. You may withdraw during the survey at any time by closing your browser. Please note that confidentiality cannot be guaranteed while data are in transit over the Internet. Non-identifying information may be used in published materials and presentations. Please remember your PARTICIPATION IS VOLUNTARY, and at any time you may decide to skip a question or not participate. The survey will take about 25 minutes to complete and will include questions about your demographic. None of the questions asked in this survey are in any way meant to suggest a dietary change is needed/required for your pet. We have no doubt that you had your pet's best interest in mind when choosing a diet. If you do have questions regarding your pet's diet, please consult your veterinarian. With your cooperation, we hope to use the information provided towards future research in companion animal nutrition. Also, your decision to participate OR not to participate in this research project will have absolutely NO consequences. There are no known risks or benefits to participant. Findings from this study will help the veterinary profession gain a better understanding of owner

perceptions related to the practice of feeding different diets.

The data collected in this study will be used to guide future research and may lead to improved nutritional recommendations from veterinarians to pet owners. Please feel free to print this page or take a photograph of this page for your records.

Findings will be published as conference abstracts presented at national and/or international animal nutrition and veterinary conferences, manuscripts published in peer-reviewed journal, articles or blog posts as well as one infographics. Information will be provided through the OVC Pet Nutrition website and social media outlets (Twitter, Facebook, Instagram). Conference presentations will be announced, a link to the published paper will be shared and a summary of results from this study will be posted on the OVC pet nutrition website. Infographics will be created for social media outlets.

Click the button below if you would like to participate in this survey.

Researcher Information: Cameron Commisso, MSc student, ccommiss@uoguelph.ca

Dr. Adronie Verbrugghe, DVM, PhD, Dip. ECVCN, Associate Professor, Royal Canin Endowed Chair in Canine and Feline Clinical Nutrition, averbrug@uoguelph.ca

This research project and MSc student stipend is supported by PPN Limited Partnership and Mitacs.

You are not waiving any legal claims, rights or remedies because of your participation in this research study. This project has been reviewed by the Research Ethics Board for compliance with federal guidelines for research involving human participants. If you have questions regarding your rights as a research participant, please contact: Manager, Research Ethics (519) 824-4120, ext. 56606

Would you like to participate in this survey?

○ Yes (1)

End of Block: Consent

Start of Block: Default Question Block

Q1 Are you the primary person that makes dietary decisions for your dog?

- ◯ Yes (1)
- ◯ No (2)

Q2 How many dogs currently live with you?

- ◯ 1 (1)
- ◯ More than 1 (please specify) (2) ____

Q3 Please only answer the following questions about one of the dogs that currently live with you, whose first letter of their name is the closet to the letter A in the alphabet.

- ◯ I agree (1)

Q4 Are there any other animals in the house?

- ◯ Yes, please specify (1) ____
- ◯ No (2)

Q5 How old is your dog? Please indicate months or years.

____

Q6 What breed is your dog?

____

Q7 What is the sex of your dog?

- ○ Male, (intact/not neutered) (1)
- ○ Male, (neutered/castrated) (2)
- ○ Female, (intact/not neutered) (3)
- ○ Female, (spayed) (4)

Q8 How long has your dog been living with you? Please indicate months or years.

____

Q9 Where did you get your dog?

- ○ Rescue/Humane Society/Shelter (1)
- ○ Breeder (2)
- ○ Friend/Family member (3)
- ○ Some was rehoming (4)
- ○ Pet Store (5)
- ○ Stray (6)
- ○ Other (please specify) (7) ____

Q10 Does your dog live indoors or outdoors?

- Exclusively indoors (1)
- Exclusively outdoors (2)
- Mostly indoors (3)
- Mostly outdoors (4)
- Half indoors, half outdoors (5)
- Other (please specify) (6) ____

Q11 Which of the following best represents your dog's role in your life? Please select all that apply.

- ▢ Companion (1)
- ▢ Sports/agility, please specify type of activity (2) ____
- ▢ Hunting, please specify the type of hunting (3) ____
- ▢ Breeding animal (4)
- ▢ Obedience (5)
- ▢ Working, please specify the type of work (6) ____
- ▢ Showing (7)
- ▢ Other, please specify (8) ____

Q12 How active is your dog?

- ◯ Over 2 hours a day (1)
- ◯ 1 hour a day (2)
- ◯ Less than 30 minutes a day (3)
- ◯ Not at all (4)

Q13 Which of the following is the best way to describe your relationship with you dog?

○ Owner (1)

○ Pet parent (2)

○ Family member (3)

○ Friend (4)

○ Companion (5)

○ Care giver (6)

○ Other, please specify (7) ____

○ I prefer not the answer (8)

Page Break

Q14 Please select and describe problems your dog has had in the last 6-12 months with any of the body systems/conditions listed

- ▢ Arthritic/bone disease (1)
- ▢ Behavioural (2)
- ▢ Cancer (3)
- ▢ Cardiovascular (heart) (4)
- ▢ Dental (5)
- ▢ Dermatologic (skin) (6)
- ▢ Ear (7)
- ▢ Endocrine (hormonal; such as diabetes thyroid, cushing) (8)
- ▢ Eye (9)
- ▢ Gastrointestinal (10)
- ▢ Kidney (11)
- ▢ Liver (12)
- ▢ Neurologic (13)
- ▢ Nutritional deficiency/toxicity (14)
- ▢ Obesity (15)
- ▢ Parasite (16)

- ☐ Pancreas (17)
- ☐ Reproductive (18)
- ☐ Trauma/injury (19)
- ☐ Urinary (20)
- ☐ Other, please specify (21) ____
- ☐ My dog has had no health concerns in the most recent 6-12 months (22)

Q15 What is your perception of your dog's behaviour, habits and appearance in the past two months?

Please drag the bar on the scale to what best represents how often your dog preforms theses behaviours.

Never	Sometimes	Often

0 10 20 30 40 50 60 70 80 90 100

How much water has your dog been drinking (1)	
How would you rate your dog's urination habits? (2)	
How would you rate your dog's bowel movement habits? (3)	
How much has your dog been eating? (4)	
How often has your dog been vomiting? (5)	
What is your impression of your dogs weight? (6)	
How is your dogs energy level? (7)	
How often does your dog groom (licking, scratching) itself? (8)	
How much has your dog been sleeping? (9)	
How has your dogs activity been? (10)	
how affectionate has your dog been towards you? (11)	

Q16 **Attention**: the following question contains images of feces. The images are from a fecal scoring chart used in many veterinary practices to grade fecal consistency. You may skip this question if you are uncomfortable with the images.

- ◯ Proceed (1)
- ◯ Skip to the next question (2)

Q17 Please select the image that you believe most closely resembles the **ideal** fecal condition for dogs.

○ 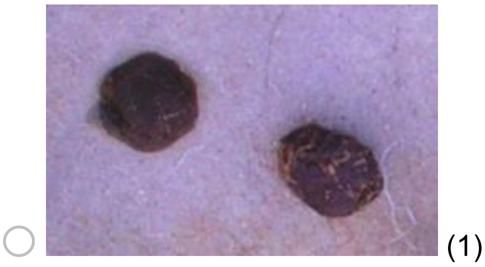 (1)

○ 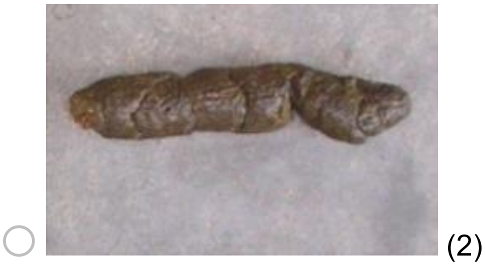 (2)

○ 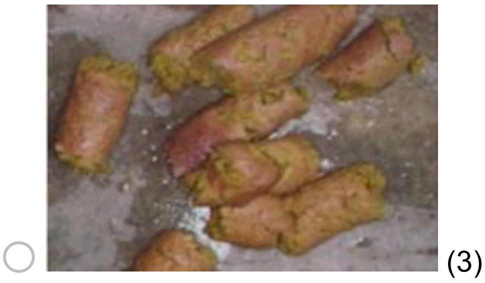 (3)

○ 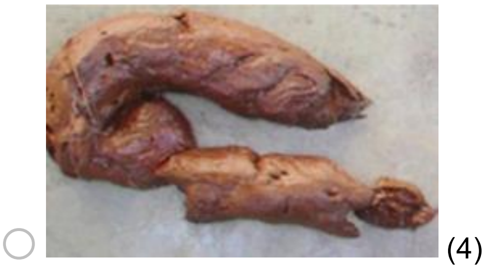 (4)

○ 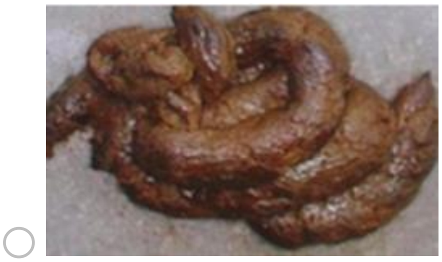 (5)

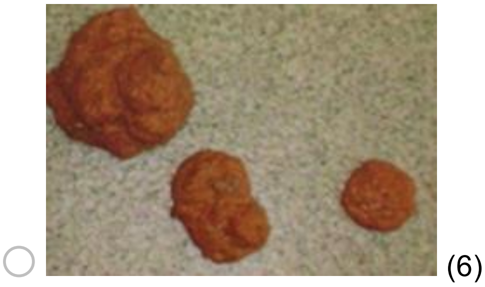 (6)

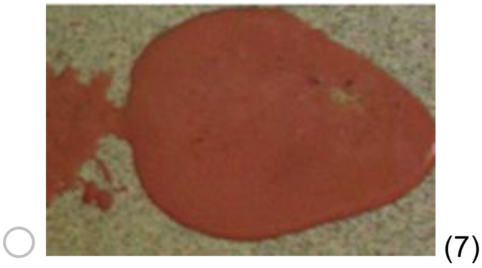 (7)

Q18 Please select the image that most closely resembles **your dog's** typical feces.

○ 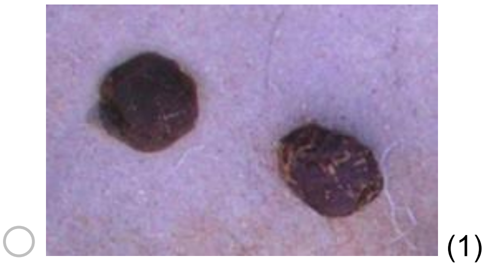 (1)

○ 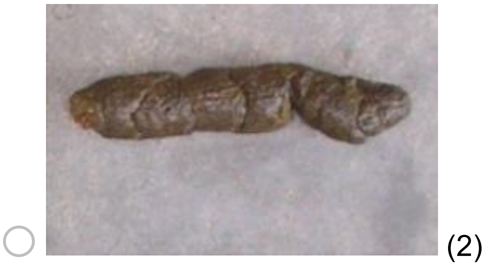 (2)

○ 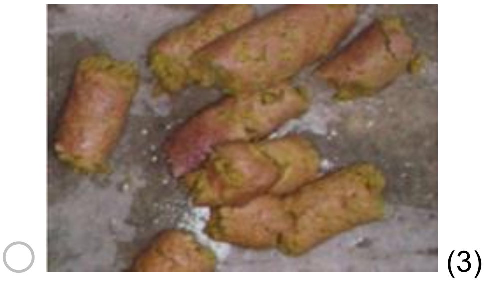 (3)

○ 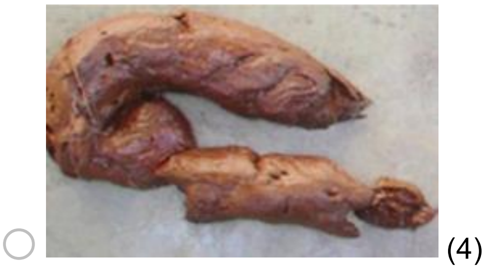 (4)

○ 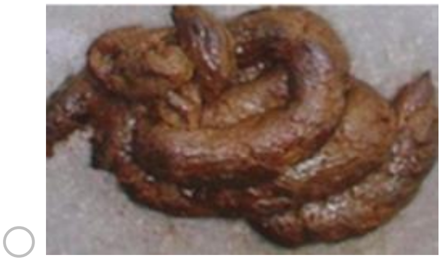 (5)

○ 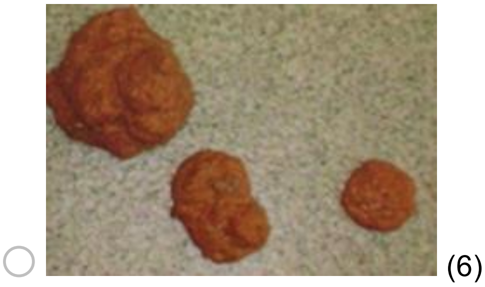 (6)

○ 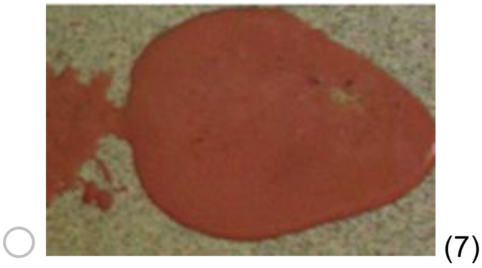 (7)

Q19 The following images are from a body condition scoring chart used in many veterinary practices to grade dog's body condition.
Please select the image that you believe most closely resembles the **ideal** body condition for a dog?

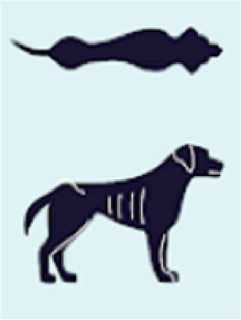
(1)

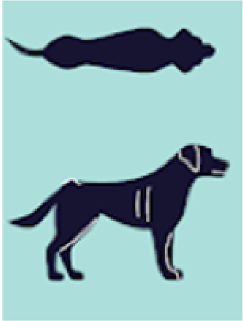
(2)

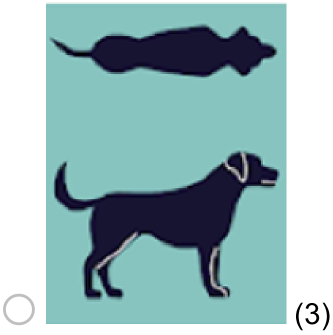
(3)

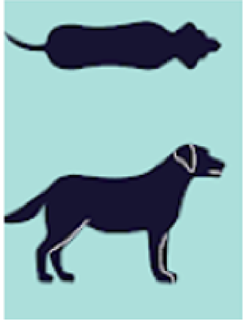
(4)

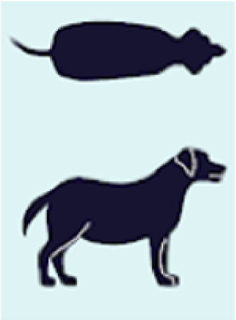 (5)

Q20 Please select the image that most closely resembles **your dog's** body condition.

○ 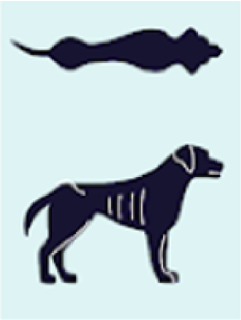 (1)

○ 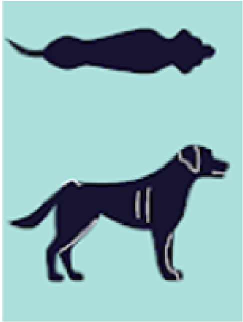 (2)

○ 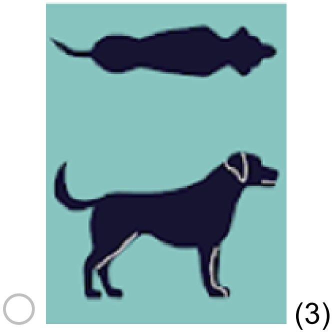 (3)

○ 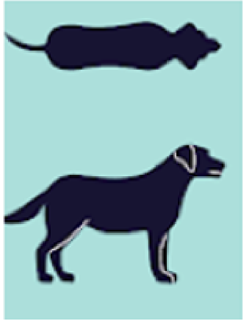 (4)

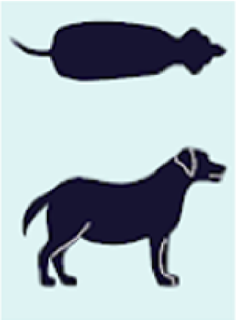 (5)

Q21 When do you take your dog to see a veterinarian? Please select all that apply.

- Only when my pet is sick (1)
- When my pet is sick and at annual checkup (2)
- Only at annual checkup (3)
- I have never taken my dog to the vet (4)
- It has been more than 1 year since I have taken my dog to the vet (5)
- Other, please specify (6) ____

Q22 When you take your dog to the vet, how often does the veterinarian discuss your dog's nutrition with you?

- Always (1)
- Sometimes (2)
- Rarely (3)
- Never (4)
- I don't remember (5)

Q23 How frequently do you want your veterinarian to discuss your dog's nutrition with you?

- Always (1)
- Sometimes (2)
- Rarely (3)
- Never (4)
- I don't know (5)

Q24 How much do you trust your veterinarians advice in regards to your dog's health? Please drag the bar on the scale to what best represents how you feel, from none at all to a great deal.

	None at all	A little	A moderate amount	A lot	A great deal
	0 10	20 30	40 50 60	70 80	90 100
General Health (excluding nutrition) (1)					
Nutritional Aspects of Health (2)					

Q25 Please select the reasons why you do not completely trust your veterinarian regarding your dog's nutrition. Please select all that apply.

- ▢ I do not think my veterinarian is knowledgeable/competent enough in terms of nutrition for my dog (1)
- ▢ I feel like my veterinarian is always trying to sell products that they carry when they make recommendations for my dog (2)
- ▢ I do not think my veterinarian listens to me or understands my needs (3)
- ▢ I do not think my veterinarian understands the needs of my dog (4)
- ▢ I completely trust my vet (5)
- ▢ Other (please specify) (6) ____

Q26 What would it take on the part of your veterinarian for you to increase your trust score regarding your dog's health?
Please rank your response in order of most to least importance from 1-5.

______ Explaining your dog's general health (excluding nutrition) in terms that can more easily be understood (1)
______ Explaining your dog's nutrition in terms that can more easily be understood (2)
______ Your veterinarian needs to show a stronger bond with your dog (3)
______ Your veterinarian needs to reduce the use of jargon (4)
______ Your veterinarian needs to better explain the cost behind treatments and the need for these costs/treatment (5)

Q27 How comfortable do you feel talking to your veterinarian about your dog's health?
Please drag the bar on the scale to what best represents how you feel, from extremely uncomfortable, neutral to extremely comfortable.

	Extremely uncomfortable	Somewhat uncomfortable	Neither comfortable nor uncomfortable	Somewhat comfortable	Extremely comfortable
	0 10	20 30	40 50 60	70 80	90 100
General health (excluding nutrition) (1)					
Nutritional Aspects of Health (2)					

Q28 What would it take on the part of your veterinarian for you to increase your comfort score related to talking to your vet about your dog's health?
Please rank your response in order of most to least importance from 1 - 5.

______ Explaining your dog's general health (excluding nutrition) in terms that can more easily be understood (1)
______ Explaining your dog's nutrition in terms that can more easily be understood (2)
______ Your veterinarian needs to show a stronger bond with your dog (3)
______ Your veterinarian needs to reduce the use of jargon (4)
______ Your veterinarian needs to better explain the cost behind treatments and the need for these costs/treatments (5)

Q29 How often do you think about your dog's nutrition?

- ○ Every time they eat (1)
- ○ Regularly but not daily (2)
- ○ Intermittently (3)
- ○ Never (4)
- ○ I am not sure how to answer this question (5)

Q30 What do you use as a primary source of information to better understand your dog's nutrition? Please select all that apply.

- ▢ Books (1)
- ▢ Dog breeder (2)
- ▢ Dog trainier (3)
- ▢ Pet store employee (4)
- ▢ Government agencies (5)
- ▢ Veterinary primary health care team (e.g. family veterinarian) (6)
- ▢ Veterinary specialist (not a nutritionist) (7)
- ▢ Board-certified veterinary nutritionist/registered veterinary technician nutrition specialist (8)
- ▢ Internet search (e.g. Google) (9)
- ▢ Pet health, food and nutrition blogs (10)
- ▢ Youtube/other health videos online (11)
- ▢ Pet health expert in the media (e.g. TV, radio, newspaper, magazine) (12)
- ▢ Social media (e.g. Facebook, Twitter, Instagram, Snapchat) (13)
- ▢ Friend/Family member (14)
- ▢ Pet food company/manufacturer (15)
- ▢ Peer-reviewed journals (16)

☐ Other, please specify (17) ____

Q31 When you see a piece of information or a recommendation about dog food or dog nutrition, do you check the qualifications of the author?

○ Yes (1)

○ No (2)

○ Sometimes (3)

○ Other, please specify (4) ____

Q32 How much do you trust each of the sources listed below for information or a recommendation about pet foods or pet nutrition?
Please rank each option in order from most important to least important on a scale from 1-7.
_______ Regulatory standards set for pet food industry (1)
_______ Family veterinarians recommending certain pet diets (2)
_______ Advice from a reputable breeder or dog trainer for your dog's nutrition (3)
_______ The messages the media portrays with regards to your dog's nutrition (4)
_______ Advice from a pet health professional who's sponsored by a certain pet food company (5)
_______ Online sources for pet nutrition (6)
_______ Pet store employees offering dog food recommendations while shopping for your pet's food (7)

Q33 How knowledgeable do you think you are about dog nutrition?

- Extremely knowledgeable (1)
- Very knowledgeable (2)
- Moderately knowledgeable (3)
- Slightly knowledgeable (4)
- Not knowledgeable at all (5)

Q34 Where do you typically purchase your pets food? Please select all that apply.

- Pet specialty store (1)
- Online retailer (2)
- Discount/mass retailers (e.g., Costco or Walmart) (3)
- Hardware store (e.g., Canadian Tire, Home Hardware) (4)
- Supermarket/grocery store (e.g., The Real Canadian Superstore, Metro, Food Basics) (5)
- Veterinary clinic/hospital (6)
- Purchase the raw products and make my own food (7)
- Others (please specify) (8) ____

Q35 What type(s) of food do you currently feed your dog? This is the food that goes in your pet's bowl or makes up the main part of his/her daily diet. Please select all that apply.

- ▢ Extruded dry/kibble pet food (1)
- ▢ Wet/canned pet food (2)
- ▢ Fresh refrigerated steam-cooked pet food (3)
- ▢ Fresh refrigerated raw per food (4)
- ▢ Freeze- dried raw food (5)
- ▢ Dehydrated or air- dried pet food (6)
- ▢ Frozen raw pet food (7)
- ▢ Home-made cooked diet (8)
- ▢ Home-made raw diet (9)
- ▢ Other, please specify (10) ____

Page Break

Q36 If you currently feed a commercial pet food product (i.e. extruded/kibble, canned/wet, fresh refrigerated steam cooked, fresh refrigerated raw, freeze-dried, dehydrated/air-dried, frozen raw), please specify the brand and product name.

____

Page Break

Q37 If you provide a raw or cooked home-made diet, please provide the recipe, the source of the recipe, as well as the quantity fed per day.

____

Page Break

Q38 How processed, preserved or raw do you consider the diet that you are currently feeding to your dog to be. Please drag the bar on the scale to what best represents what you think, from very little, neutral, to very much.

	0 10 20 30 40 50 60 70 80 90 100
Processed (1)	
Preserved (2)	
Raw (3)	

Q39 How long has your pet been on their current diet?

- ○ Months (1) ____
- ○ Years (2) ____
- ○ Other, please specify (3) ____

Q40 In the past 4-6 months, have you changed your pet's diet?

- ○ Yes (1)
- ○ No (2)
- ○ I don't remember (3)

Page Break

Q41 If you changed your pet’s diet in the past 4-6 months, please explain what type of change and why you chose to do so.

____

Page Break

Q42 Do you have plans to change your dog's diet in the next 4-6 months?

- ○ Yes (1)
- ○ No (2)
- ○ Not sure at this time (3)

Page Break

Q43 If you plan to change your pet's diet in the next 4-6 months, please explain what type of diet you would switch to and why.

____

Page Break

Q44 Please indicate on the scale how satisfied are you with your dog's current diet? Slide the scale from extremely dissatisfied, neutral, to extremely satisfied

	Extremely dissatisfied	Somewhat dissatisfied	Neither satisfied nor dissatisfied	Somewhat satisfied	Extremely satisfied

0 10 20 30 40 50 60 70 80 90 100

How satisfied are you with your dog's diet? (1)	

Q45 Thinking about the food you currently feed your dog, how would you rate it in terms of health benefits?

○ No health benefits (1)

○ Limited health benefits (2)

○ Average health benefits (3)

○ Above average health benefits (4)

○ Unsure (5)

Q46 Please explain your reasoning for the health property rating provided in the previous question

____

Q47 Regarding each of the conditions listed below, do you think your dog is at risk right now for developing the condition?

	Yes (1)	Unsure (2)	No (3)
Arthritis/bone disease (1)	○	○	○
Behavioural (2)	○	○	○
Cancer (3)	○	○	○
Cardiovascular/heart disease (4)	○	○	○
Dental disease (5)	○	○	○
Dermatologic (skin and ear) (6)	○	○	○
Endocrine/hormonal disease (e.g. diabetes, thyroid, cushings) (7)	○	○	○
Eye Disease (8)	○	○	○
Gastrointestinal Disease (9)	○	○	○
Kidney Disease (10)	○	○	○
Liver Disease (11)	○	○	○
Nutritional deficiency/toxicity (12)	○	○	○
Obesity (13)	○	○	○
Parasitic disease (14)	○	○	○
Pancreatic disease (15)	○	○	○
Reproductive disease (16)	○	○	○

Trauma/injury (17)	○	○	○
Urinary disease (18)	○	○	○

Q48 What factors do you think are important when selecting food for your dog? Please select all that apply.

- ☐ Benefits to skin and coat health (1)
- ☐ Complete and balanced (2)
- ☐ Convenient to feed (3)
- ☐ Convenient to purchase (4)
- ☐ Digestibility (5)
- ☐ Easy access package/storage capability (6)
- ☐ Environmentally friendly/sustainable (7)
- ☐ Exclusion of specify ingredients, please specify (8) ____
- ☐ Fecal consistency (9)
- ☐ Flavour/palatability (10)
- ☐ Fresh (11)
- ☐ Gluten free (12)
- ☐ Grain free (13)
- ☐ Price value (14)
- ☐ Had good internet reviews (15)
- ☐ High quality ingredients (16)

- ☐ Homemade (17)
- ☐ Inclusion of specific ingredients, please specify (18) ____
- ☐ Meat first on ingredient list (19)
- ☐ Minimally processed (20)
- ☐ Most similar to ancestral diet (21)
- ☐ Natural/holistic (22)
- ☐ No artificial additives or preservatives (23)
- ☐ Non-GMO ingredients (24)
- ☐ Organic (25)
- ☐ Preference of my pet (26)
- ☐ Prevention of aliment/condition, please specify (27) ____
- ☐ Raw ingredients (28)
- ☐ Recommend by breeder (29)
- ☐ Recommended by board-certified veterinary nutritionist(s) (30)
- ☐ Recommended by friend/family member (31)
- ☐ Recommended by pet food company (32)
- ☐ Recommended by pet store/pet food store/grocery store/department store (33)

- ☐ Recommended by rescue/humane society/shelter (34)
- ☐ Recommended by the pet's previous pet guardian (35)
- ☐ Recommended by veterinarian(s) (NOT board-certified veterinary nutritionist) (36)
- ☐ Recommended by veterinary technician(s)/nurse(s) (37)
- ☐ Reputation of the company (38)
- ☐ Safe to feed and of little risk to my family (39)
- ☐ Treatment of ailment/condition, please specify (40) ____
- ☐ With grains (41)
- ☐ Other, please specify (42) ____

Q49 At this time, do you feed your dog additional vitamins/supplements?

- ○ Yes (1)
- ○ No (2)

Page Break

Q50 If you feed your dog additional vitamins/supplements. Please specify brand, product name and how you determine quantity to give.

____

Page Break

Q51 If you feed additional vitamins/supplements, what are your reasons for doing so? Please select all that apply.

- ▢ Recommended by veterinarian(s) (NOT board-certified veterinary nutritionists) (1)
- ▢ Recommended by veterinary technician(s)/nurse(s) (2)
- ▢ Recommended by board-certified veterinary nutritionists (3)
- ▢ Recommended by rescue/humane society/shelter (4)
- ▢ Recommended by breeder (5)
- ▢ Recommended by pet store/pet food store/grocery store/department store (6)
- ▢ Recommended by my pet's previous pet guardian (7)
- ▢ Recommended by friend/family member (8)
- ▢ Recommended in books/blogs/social media post (9)
- ▢ Recommended by pet food company (10)
- ▢ To ensure that my pet's current diet is complete and balanced (11)
- ▢ Believe that additional vitamins/supplements will improve my pet's health (12)
- ▢ Other, please specify (13) ____

Q52 What degree of risk of food-borne illness due to bacterial contamination do you believe is associated with feeding your dog either commercial pet food or a homemade pet food?   Slide the scale from low risk to high risk.

	0 10 20 30 40 50 60 70 80 90 100
Commercial Pet Food (1)	
Homemade Pet Food (2)	

Q53 Has your veterinarian ever discussed the risks of food-borne illness due to bacterial contamination associated with feeding your pet?

- ○ Yes (1)
- ○ No (2)
- ○ I can't remember (3)

Q54 How often do you wash your dog's food and water bowls?

- ○ Never (1)
- ○ Once a month (2)
- ○ Twice a month (3)
- ○ Once every week (4)
- ○ Once every day (5)
- ○ Other (please specify) (6) ____

Q55 Do you wash your hands with soap and water after handling your pet's food?

- Never (1)
- Sometimes (2)
- About half the time (3)
- Most of the time (4)
- Always (5)

Q56 Do you wash your hands after touching surfaces (counters, the refrigerator, utensils, cutting boards, the microwave) that came into contact with your pet's food?

- Never (1)
- Sometimes (2)
- About half the time (3)
- Most of the time (4)
- Always (5)

Q57 Do you clean all surfaces that came into contact with your pet's food using a disinfectant?

- Never (1)
- Sometimes (2)
- About half the time (3)
- Most of the time (4)
- Always (5)

Q58 Do you store your pet's food in a separate area from food used for human consumption?

- Never (1)
- Sometimes (2)
- About half the time (3)
- Most of the time (4)
- Always (5)

Q59 Do you feed your pet's food in a separate area from where human food consumption occurs?

- Never (1)
- Sometimes (2)
- Always (3)

Q60 Do you allow you dog to lick your face or kiss you?

- Yes (1)
- No (2)
- On occasion (3)

Q61 If your dog defecates in the yard, how quickly do you pick it up?

- ◯ Right after they go (1)
- ◯ Once a day (2)
- ◯ Once every few days (3)
- ◯ Once a week (4)
- ◯ Monthly (5)
- ◯ Other, please specify (6) ____

Page Break

Q62 When evaluating how much you trust pet food companies, which of the following considerations is most important?
Please rank your response in order of most to least importance from 1-7.

	Cooked pet food (extruded dry/kibble pet food, wet canned pet food) (4)	Non-cooked pet food products (fresh raw, frozen raw, dehydrated/air-dried or freeze-dried pet food) (5)
Pet food formulation (1)		
Ingredient sourcing (2)		
Quality control (3)		
Pet food safety (4)		
Package labeling (5)		
Feeding guidelines (6)		
Putting pet health and wellbeing first (7)		

Q63 What would it take to increase your trust in pet food in the following areas of pet health and nutrition?

Please rank your response in order of most to least importance from 1-7.

	Cooked pet food (extruded dry/kibble pet food, wet canned pet food) (1)	Non-cooked pet food (fresh raw, frozen raw, dehydrated/air-dried or freeze-dried pet food) (2)
More information regarding the pet food formulation (1)		
More information regarding ingredient sourcing (2)		
Improved quality control (3)		
Information regarding pet food safety/regulations (4)		
Improved package labeling (5)		
Improved feeding guidelines (6)		
Choosing a company that puts pet health and wellbeing first (7)		

Q64 To which extent do you agree with the following statements? Please drag the bar on the scale to what best represents how you feel, from strongly disagree, neutral and strongly agree.

	Strongly Disagree	Neutral	Strongly Agree
	0 10 20 30 40	50 60	70 80 90 100
I believe nutrition plays an important role in my dog's health (1)			
The label and list of ingredients are important when selecting my dog's food (2)			
Good quality extruded dry/kibble or wet/canned pet foods contain ALL the nutrition my pet needs (3)			
I believe a homemade diet may provide better nutrition for my pet than extruded dry/kibble or wet/canned pet foods (4)			
I believe a raw meat diet may provide better nutrition for my pet than extruded dry/kibble or wet/canned pet foods (5)			
Dogs are carnivores so they need a meat-based diet (6)			
Grains such as wheat, corn, rice, etc. are good sources of nutrition for dogs (7)			
Cooking destroys nutrients in pet foods (8)			
Dogs need to eat a variety of foods and this should vary from day to day (9)			

Page Break

Q65 How often do you think about your own nutrition?

- ◯ Every time you eat (1)
- ◯ Regularly but not daily (2)
- ◯ Intermittently (3)
- ◯ Never (4)
- ◯ I am unsure of how to answer this question (5)
- ◯ I prefer not to answer (6)

Q66 How healthy do you think your diet is?

- ◯ Very healthy (1)
- ◯ Moderately healthy (2)
- ◯ Neither healthy nor unhealthy (3)
- ◯ Moderately unhealthy (4)
- ◯ Prefer not to answer (5)

Q67 Do you currently eat a diet that is considered:

- ○ Carnivorous (only animal ingredients, no plant ingredients) (1)
- ○ Omnivorous (both animal and plant ingredients) (2)
- ○ Pescatarian (plant ingredients, but also fish and other seafood) (3)
- ○ Vegetarian (plant ingredients, but also eggs and dairy) (4)
- ○ Vegan (strictly plant ingredients) (5)
- ○ Other, please specify (6) ____

Q68 Do you currently follow a specific human food trend and/or do you have dietary restrictions (e.g. paleo, keto, grain-free, gluten free, sugar-free, nut-free, plant-based, natural, organic etc.)?

- ○ Yes (1)
- ○ No (2)
- ○ Prefer not to answer (3)

Page Break

Q69 If you currently follow a human food trend and/or you have dietary restrictions, please specify your diet and explain your choice.

____

Page Break

Q70 Are you the person who usually prepares the meals for yourself and/or family members within your household

- Yes (1)
- No (2)

Q71 On an average day, how much time do you spend to prepare your meals—preparing food and beverages, serving the food, and cleaning up afterwards

- Less than 30 minutes (1)
- 30 minutes to an hour (2)
- 1 to 2 hours (3)
- 2 hours or more (4)

Q72 Do you spend a few hours on a specific day of the week to prepare all of your meals for the upcoming week and portion them into containers that are either refrigerated or frozen (meal prepping) or preparing a couple of different types of foods in large batches and storing them separately in the fridge for easy access throughout the week (batch cooking)?

- Yes (1)
- No (2)

Page Break

Q73 For how many days of the week do you meal prep or batch cook for yourself and/or family members (not pets)?

- 1 to 2 days a week (1)
- 3 to 4 days a week (2)
- 4 to 5 days a week (3)
- 5 to 6 days a week (4)
- Every day of the week (5)
- I do not batch cook (6)

Q74 How many times in the past week, did you purchase prepared food from a deli, carry-out, delivery food, restaurant or fast food for yourself?

- I did not buy any prepared food this week (1)
- 1 to 2 times a week (2)
- 3 to 4 times a week (3)
- 5 to 6 times a week (4)
- Every day this week (5)

Q75 How much knowledge do you think you have about human nutrition?

- Extremely knowledgeable (1)
- Moderately knowledgeable (2)
- Slightly knowledgeable (3)
- Not knowledgeable at all (4)

Q76 How confident are you in knowing how to make changes to your own diet?

- Strongly confident (1)
- Moderately confident (2)
- Neither confident nor unconfident (3)
- Moderately unconfident (4)
- Strongly unconfident (5)

Q77 What do you use as primary source of information to better understand your own nutrition? Please select all that apply.

- ▢ Family health team (e.g. family physician, nurse practitioner) (1)
- ▢ Medical specialist (not a nutrition specialist) (2)
- ▢ Registered dietician/certified clinical nutritionist/certified nutrition specialist (3)
- ▢ Government agencies (4)
- ▢ Peer-reviewed journal articles (5)
- ▢ Internet search (e.g., Google) (6)
- ▢ Health, food, nutrition blogs (7)
- ▢ YouTube/other health videos online (8)
- ▢ Food expert on the media (e.g. TV, radio commercials, magazines) (9)
- ▢ Books (10)
- ▢ Social media sites (e.g. Facebook, Twitter, Instagram, Snapchat) (11)
- ▢ Friend/family member (12)
- ▢ Personal trainer (13)
- ▢ Food company/manufacturer (14)
- ▢ Grocery store (15)
- ▢ Other, please specify (16) ____

Q78 When you see a piece of information or a recommendation about human food or human nutrition, do you check the qualifications of the author?

◯ Yes (1)

◯ No (2)

◯ Sometimes (3)

◯ Other, please specify (4) ____

Q79 How much do you trust each of the sources listed below for information or a recommendation about human foods or human nutrition? Please drag the bar on the scale to what best represents how you feel, from extreme distrust, neutral to extreme trust

	Extreme distrust	Neutral	Extreme trust
	0 10 20 30 40 50 60 70 80 90 100		
Regulatory standards set for human food industry (1)			
Family doctors recommending certain diets (2)			
Advice from a personal trainer for your own nutrition (3)			
The messages the media portrays with regards to your own nutrition (4)			
Advice from a health professional who's sponsored by a certain food company (5)			
Store clerks offering diet recommendations while shopping for your own food (6)			
Online sources for your own nutrition (7)			

Page Break

Q80 Where is your permanent residence?

○ Canada (1)

○ USA (2)

○ Other, please specify (3) ____

Q81 Please select which province or territory you're from

▼ Alberta (1) ... Yukon (13)

Q82 Please select which state you're from

▼ Alabama (1) ... Wyoming (50)

Q83 How old are you?

○ Years (1) ____

Q84 What is your self-identified gender?

○ Male (1)

○ Female (2)

○ Non-binary / third gender (3)

○ My gender identity is not listed above (4)

○ Prefer not to say (5)

Q85 Which of the following best describes your HIGHEST level of education?

- Some high school (1)
- Completed high school (2)
- Some college/university (3)
- Apprenticeship training and trades (4)
- Completed college/university (5)
- Some graduate education (6)
- Completed graduate education (7)
- Professional degrees (8)
- Choose not to respond (9)

Q86 Are you currently employed or have you been employed in the pet care industry?

- Yes (1)
- No (2)

Q87 What is your occupation?

____

Q88 Please select a range that includes your current household income.

- Less than $19,999 (1)
- $20,000-$39,000 (2)
- $40,000-$59,000 (3)
- $60,000-$79,000 (4)
- $80,000-$90,000 (5)
- $90,000-$100,000 (6)
- Greater than $100,000 (7)
- I would prefer not to answer (8)

Q89 A FINAL QUESTION, DO YOU WANT TO WIN A PRIZE? Are you interested in being entered in a draw to win a $30 CAD Amazon gift card? 1 in 100 chance to win!

- Yes (1)
- No (2)

End of Block: Default Question Block
