# Supplementary material for: Insights into the influence of dog and guardian demographics, nutrition, and relationship on raw feeding practices
Source: Front Vet Sci. 2026 May 20;13:1793754. doi: 10.3389/fvets.2026.1793754 (PMC13231891; doi:10.3389/fvets.2026.1793754)
Supplement: Supplementary file 2 [file Table_1.PDF]

Supplementary Material

Table S1 Univariate logistic regression analyses assessing guardianship, lifestyle, and environmental characteristics of dogs fed raw versus cooked diets.

	Raw – n (%)	Cooked – n (%)	OR (95% CI)*	P-value†
**Duration of symbiosis (n=427)**				
Less than 12 months	23 (16.5)	52 (18.1)	Ref	
12-36 months	63 (45.3)	124 (43.1)	1.15 (0.65-2.05)	0.638
37-60 months	23 (16.5)	42 (14.6)	1.24 (0.61-2.51)	0.554
61-84 months	11 (7.9)	28 (9.7)	0.89 (0.38-2.08)	0.785
Greater than 84 months	19 (13.7)	42 (14.6)	1.02 (0.49-2.13)	0.952
**Activity (n=431)**				
Less than 30 minutes a day	11 (7.7)	26 (9.0)	Ref	
1 hour a day	60 (42.3)	134 (46.4)	1.06 (0.49-2.28)	0.885
Over 2 hours a day	71 (50.0)	129 (44.6)	1.30 (0.61-2.79)	0.499
**Housing (n=432)**				
Exclusively indoors	91 (64.5)	202 (69.4)	Ref	
Mostly indoors	46 (32.6)	77 (26.5)	1.33 (0.85-2.06)	0.210
Half indoors, half outdoors	4 (2.8)	12 (4.1)	0.74 (0.23-2.36)	0.610
**Presence of other pets in household (n=433)**				
Single-pet household	60 (42.3)	132 (45.4)	Ref	
Multi-pet household	82 (57.7)	159 (54.6)	1.14 (0.76-1.70)	0.541
**Presence of cats in multi-pet households (n=240)**				
No cats	36 (43.9)	65 (41.1)	Ref	
With cats	46 (56.1)	93 (58.9)	0.89 (0.52-1.53)	0.681

*Confidence intervals (CI) and Odds ratios (OR) refer to the probability of being a member of the raw group; †Significance of difference compared with reference (Ref) category

Table S2 Univariate logistic regression analyses assessing characteristics of raw versus cooked canine diets.

	Raw – n (%)	Cooked – n (%)	OR (95% CI)*	P-value†
**Duration on diet (n=407)**				
Less than 12 months	36 (28.3)	103 (36.8)	Ref	
12-35 months	48 (37.8)	92 (32.9)	1.49 (0.89-2.50)	0.128
36-71 months	26 (20.5)	60 (21.4)	1.24 (0.68-2.25)	0.480
Greater than 71 months	17 (13.4)	25 (8.9)	1.95 (0.94-4.01)	0.071
**Any diet change in the past 4-6 months (n=421)**				
Yes	38 (28.1)	87 (30.4)	0.90 (0.57-1.41)	0.634
No	97 (71.9)	199 (69.6)	Ref	
**Intention to modify the diet in the next 4-6 months (n=419)**				
Yes	7 (5.2)	31 (10.9)	0.49 (0.21-1.15)	0.102
Not sure at this time	37 (27.4)	56 (19.7)	1.43 (0.88-2.32)	0.147
No	91 (67.4)	197 (69.4)	Ref	
**Purchasing location: (n=432)**				

**Pet specialty store**						
Selected	125	(88.0)	209	(72.1)	2.85 (1.62-5.03)	<0.001
Not selected	17	(12.0)	81	(27.9)	Ref	
**Online retailer**						
Selected	15	(10.6)	29	(10.0)	1.06 (0.55-2.05)	0.856
Not selected	127	(89.4)	261	(90.0)	Ref	
**Discount/mass retailer**						
Selected	8	(5.6)	32	(11.0)	0.48 (0.22-1.07)	0.074
Not selected	134	(94.4)	258	(89.0)	Ref	
**Supermarket/grocery store**						
Selected	3	(2.1)	20	(6.9)	0.29 (0.09-1.00)	0.050
Not selected	139	(97.9)	270	(93.1)	Ref	
**Veterinary clinic/hospital**						
Selected	13	(9.2)	64	(22.1)	0.36 (0.19-0.67)	0.001
Not selected	129	(90.8)	226	(77.9)	Ref	
**Purchase raw products and make my own food**						
Selected	42	(29.6)	19	(6.6)	5.99 (3.33-10.79)	<0.001
Not selected	100	(70.4)	271	(93.4)	Ref	
**Provision of supplements (n=393)**						
Yes	84	(67.7)	105	(39.0)	3.28 (2.09-5.14)	<0.001
No	40	(32.3)	164	(61.0)	Ref	
**Types of supplements: (n=174)**						
**Joint supplements**						
Mentioned	27	(35.5)	36	(36.7)	0.95 (0.51-1.77)	0.869
Not mentioned	49	(64.5)	62	(63.3)	Ref	
**Fish oil**						
Mentioned	31	(40.8)	20	(20.4)	2.69 (1.37-5.26)	0.004
Not mentioned	45	(59.2)	78	(79.6)	Ref	
**Probiotics**						
Mentioned	17	(22.4)	24	(24.5)	0.89 (0.44-1.81)	0.744
Not mentioned	59	(77.6)	74	(75.5)	Ref	
**Multi-vitamin/mineral mix**						
Mentioned	9	(11.8)	6	(6.1)	2.06 (0.70-6.06)	0.190
Not mentioned	67	(88.2)	92	(93.9)	Ref	
**Reason for supplement provision: (n=188)**						
**Recommended by veterinarians (not board-certified veterinary nutritionists)**						
Selected	25	(30.5)	50	(47.2)	0.49 (0.27-0.90)	0.021
Not selected	57	(69.5)	56	(52.8)	Ref	
**Recommended by veterinary technician/nurse**						
Selected	6	(7.3)	13	(12.3)	0.57 (0.21-1.56)	0.269
Not selected	76	(92.7)	93	(87.7)	Ref	
**Recommended by board-certified veterinary nutritionist**						
Selected	12	(14.6)	13	(12.3)	1.23 (0.53-2.85)	0.635
Not selected	70	(85.4)	93	(87.7)	Ref	
**Recommended by breeder**						
Selected	14	(17.1)	10	(9.4)	1.98 (0.83-4.71)	0.124
Not selected	68	(82.9)	96	(90.6)	Ref	
**Recommended by pet store/pet food store/grocery store/department store**						
Selected	8	(9.8)	6	(5.7)	1.80 (0.60-5.42)	0.294
Not selected	74	(90.2)	100	(94.3)	Ref	

**Recommended by friend/family member**						
Selected	6	(7.3)	13	(12.3)	0.57 (0.21-1.56)	0.269
Not selected	76	(92.7)	93	(87.7)	Ref	
**Recommended in books/blogs/social media post**						
Selected	14	(17.1)	12	(11.3)	1.61 (0.70-3.71)	0.260
Not selected	68	(82.9)	94	(88.7)	Ref	
**Recommended by pet food company**						
Selected	12	(14.6)	1	(0.9)	18.00 (2.29-141.55)	0.006
Not selected	70	(85.4)	105	(99.1)	Ref	
**To ensure my pet's current diet is complete and balanced**						
Selected	47	(57.3)	24	(22.6)	4.59 (2.44-8.63)	<0.001
Not selected	35	(42.7)	82	(77.4)	Ref	
**I believe that additional vitamins/supplements will improve my pet's health**						
Selected	46	(56.1)	61	(57.5)	0.94 (0.53-1.69)	0.842
Not selected	36	(43.9)	45	(42.5)	Ref	

*Confidence intervals (CI) and Odds ratios (OR) refer to the probability of being a member of the raw group; †Significance of difference compared with reference (Ref) category

Table S3 Univariate logistic regression analyses exploring the relationship between feeding practice (raw or cooked) and dog guardians' pet food choices and perceived knowledge of canine nutrition.

	Raw – n (%)		Cooked - n (%)		OR (95% CI)*	P-value†
**Frequency of thinking about dog's nutrition (n=426)**						
Every time they eat	68	(48.6)	97	(33.9)	2.51 (1.23-5.12)	0.011
Regularly but not daily	60	(42.9)	146	(51.0)	1.47 (0.73-2.99)	0.283
Intermittently	12	(8.6)	43	(15.0)	Ref	
**Perceived knowledge of canine nutrition (n=431)**						
Extremely	13	(9.2)	24	(8.3)	2.26 (0.90-5.68)	0.084
Very	43	(30.3)	65	(22.5)	2.76 (1.32-5.77)	0.007
Moderately	72	(50.7)	141	(48.8)	2.13 (1.07-4.25)	0.032
Slightly	12	(8.5)	50	(17.3)	Ref	
Not at all	2	(1.4)	9	(3.1)	0.93 (0.18-4.85)	0.927
**Satisfaction with current diet (n=393)**						
Extremely satisfied	85	(69.1)	165	(61.1)	1.87 (0.82-4.26)	0.138
Somewhat satisfied	27	(22.0)	65	(24.1)	1.51 (0.61-3.71)	0.374
Neither satisfied nor dissatisfied	8	(6.5)	29	(10.7)	Ref	
Dissatisfied	3	(2.4)	11	(4.1)	0.99 (0.22-4.42)	0.988
**Processed (n=381)**						
Very much	30	(27.0)	159	(58.9)	0.53 (0.30-0.93)	0.027
Neutral	32	(28.8)	90	(33.3)	Ref	
Very little	49	(44.1)	21	(7.8)	6.56 (3.42-12.59)	<0.001
**Preserved (n=343)**						
Very much	22	(22.4)	107	(43.7)	0.72 (0.38-1.34)	0.296
Neutral	27	(27.6)	94	(38.4)	Ref	
Very little	49	(50.0)	44	(18.0)	3.88 (2.15-7.00)	<0.001
**Raw (n=247)**						
Very much	70	(56.0)	6	(4.9)	8.26 (2.92-23.38)	<0.001
Neutral	24	(19.2)	17	(13.9)	Ref	
Very little	31	(24.8)	99	(81.2)	0.22 (0.11-0.47)	<0.001

*Confidence intervals (CI) and Odds ratios (OR) refer to the probability of being a member of the raw group; †Significance of difference compared with reference (Ref) category

Table S4 Univariate logistic regression analyses of factors influencing pet food selection in dog guardians feeding raw or cooked diets.

	Raw – n (%)		Cooked – n (%)		OR (95 CI)[a]	P-value[b]
**Important factors during pet food selection: (n=394)**						
**Benefits to skin and coat health**						
Selected	94	(75.2)	187	(69.5)	1.33 (0.82-2.15)	0.246
Not selected	31	(24.8)	82	(30.5)	Ref	
**Complete and balanced**						
Selected	106	(84.8)	242	(90.0)	0.62 (0.33-1.17)	0.140
Not selected	19	(15.2)	27	(10.0)	Ref	
**Convenient to feed**						
Selected	34	(27.2)	109	(40.5)	0.55 (0.35-0.87)	0.011
Not selected	91	(72.8)	160	(59.5)	Ref	
**Convenient to purchase**						
Selected	35	(42.7)	100	(37.2)	0.66 (0.41-1.04)	0.075
Not selected	90	(82.3)	169	(62.8)	Ref	
**Digestibility**						
Selected	88	(70.4)	177	(65.8)	1.24 (0.78-1.96)	0.365
Not selected	37	(29.6)	92	(34.2)	Ref	
**Easy access package/storage capability**						
Selected	24	(19.2)	49	(18.2)	1.07 (0.62-1.84)	0.815
Not selected	101	(80.8)	220	(81.8)	Ref	
**Environmentally friendly/sustainable**						
Selected	37	(29.6)	46	(17.1)	2.04 (1.24-3.36)	0.005
Not selected	88	(70.4)	223	(82.9)	Ref	
**Exclusion of specific ingredients**						
Selected	35	(28.0)	58	(21.6)	1.42 (0.87-2.30)	0.162
Not selected	90	(72.0)	211	(78.4)	Ref	
**Fecal consistency**						
Selected	80	(64.0)	146	(54.3)	1.50 (0.97-2.32)	0.070
Not selected	45	(36.0)	123	(45.7)	Ref	
**Flavour/palatability**						
Selected	49	(39.2)	126	(46.8)	0.73 (0.48-1.13)	0.156
Not selected	76	(60.8)	143	(53.2)	Ref	
**Fresh**						
Selected	53	(42.4)	46	(17.1)	3.57 (2.22-5.74)	<0.001
Not selected	72	(57.6)	223	(82.9)	Ref	
**Gluten-free**						
Selected	8	(6.4)	9	(3.3)	1.98 (0.74-5.25)	0.172
Not selected	117	(93.6)	260	(96.7)	Ref	
**Grain-free**						
Selected	31	(24.8)	28	(10.4)	2.84 (1.62-4.99)	<0.001

Not selected	94	(75.2)	241	(89.6)	Ref	
**Price value**						
Selected	28	(22.4)	93	(34.6)	0.55 (0.34-0.89)	0.016
Not selected	97	(77.6)	176	(65.4)	Ref	
**Had good internet reviews**						
Selected	11	(8.8)	33	(12.3)	0.69 (0.34-1.42)	0.311
Not selected	114	(91.2)	236	(87.7)	Ref	
**High quality ingredients**						
Selected	105	(84.0)	174	(64.7)	2.87 (1.67-4.92)	<0.001
Not selected	20	(16.0)	95	(35.3)	Ref	
**Homemade**						
Selected	14	(11.2)	11	(4.1)	2.96 (1.30-6.72)	0.010
Not selected	111	(88.8)	258	(95.9)	Ref	
**Inclusion of specific ingredients**						
Selected	9	(7.2)	22	(8.2)	0.87 (0.39-1.95)	0.737
Not selected	116	(92.8)	247	(91.8)	Ref	
**Meat first on ingredient list**						
Selected	79	(63.2)	112	(41.6)	2.41 (1.56-3.73)	<0.001
Not selected	46	(36.8)	157	(58.4)	Ref	
**Minimally processed**						
Selected	69	(55.2)	65	(24.2)	3.87 (2.47-6.06)	<0.001
Not selected	56	(44.8)	204	(75.8)	Ref	
**Most similar to ancestral diet**						
Selected	36	(28.8)	13	(4.8)	7.97 (4.04-15.70)	<0.001
Not selected	89	(71.2)	256	(95.2)	Ref	
**Natural/holistic**						
Selected	29	(23.2)	14	(5.2)	5.50 (2.79-10.86)	<0.001
Not selected	96	(76.8)	255	(94.8)	Ref	
**No artificial additives or preservatives**						
Selected	50	(40.0)	46	(17.1)	3.23 (2.00-5.21)	<0.001
Not selected	75	(60.0)	223	(82.9)	Ref	
**Non-GMO ingredients**						
Selected	31	(24.8)	24	(8.9)	3.37 (1.88-6.03)	<0.001
Not selected	94	(75.2)	245	(91.1)	Ref	
**Organic**						
Selected	17	(13.6)	13	(4.8)	3.10 (1.46-6.60)	0.003
Not selected	108	(86.4)	256	(95.2)	Ref	
**Preference of my pet**						
Selected	50	(40.0)	109	(40.5)	0.98 (0.64-1.51)	0.922
Not selected	75	(60.0)	160	(59.5)	Ref	
**Prevention of ailment/condition**						
Selected	12	(9.6)	33	(12.3)	0.76 (0.38-1.53)	0.440
Not selected	113	(90.4)	236	(87.7)	Ref	
**Raw ingredients**						

Selected	76	(60.8)	8	(3.0)	50.60 (22.97-111.48)	<0.001
Not selected	49	(39.2)	261	(97.0)	Ref	
**Recommend by breeder**						
Selected	21	(16.8)	33	(12.3)	1.44 (0.80-2.62)	0.225
Not selected	104	(83.2)	236	(87.7)	Ref	
**Recommended by board-certified veterinary nutritionist**						
Selected	32	(25.6)	104	(38.7)	0.55 (0.34-0.87)	0.012
Not selected	93	(74.4)	165	(61.3)	Ref	
**Recommended by friend/family member**						
Selected	15	(12.0)	16	(5.9)	2.16 (1.03-4.52)	0.042
Not selected	110	(88.0)	253	(94.1)	Ref	
**Recommended by pet store/pet food store/grocery store/department store**						
Selected	7	(5.6)	14	(5.2)	1.08 (0.43-2.75)	0.871
Not selected	118	(94.4)	255	(94.8)	Ref	
**Recommended by rescue/humane society/shelter**						
Selected	5	(4.0)	11	(4.1)	0.98 (0.33-2.88)	0.967
Not selected	120	(96.0)	258	(95.9)	Ref	
**Recommended by the pet's previous guardian**						
Selected	1	(0.8)	11	(4.1)	0.19 (0.02-1.48)	0.113
Not selected	124	(99.2)	258	(95.9)	Ref	
**Recommended by veterinarians (not board-certified veterinary nutritionist)**						
Selected	13	(10.4)	92	(34.2)	0.22 (0.12-0.42)	<0.001
Not selected	112	(89.6)	177	(65.8)	Ref	
**Recommended by veterinary technician/nurse**						
Selected	9	(7.2)	52	(19.3)	0.32 (0.15-0.68)	0.003
Not selected	116	(92.7)	217	(80.7)	Ref	
**Reputation of the company**						
Selected	49	(39.2)	110	(40.9)	0.93 (0.60-1.44)	0.750
Not selected	76	(60.8)	159	(59.1)	Ref	
**Safe to feed and of little risk to my family**						
Selected	34	(27.2)	66	(24.5)	1.15 (0.71-1.86)	0.572
Not selected	91	(72.8)	203	(75.5)	Ref	
**Treatment of ailment/condition**						
Selected	3	(2.4)	17	(6.3)	0.37 (0.11-1.27)	0.112
Not selected	122	(97.6)	252	(93.7)	Ref	
**With grains**						
Selected	16	(12.8)	56	(20.8)	0.56 (0.31-1.02)	0.058
Not selected	109	(87.2)	213	(79.2)	Ref	

*Confidence intervals (CI) and Odds ratios (OR) refer to the probability of being a member of the raw group; †Significance of difference compared with reference (Ref) category

Table S5 Univariate logistic regression analyses of nine statements that assess the beliefs and considerations of dog guardians feeding raw or cooked diets regarding canine nutrition.

	Raw – n (%)	Cooked – n (%)	OR (95 CI)[a]	P-value[b]
**The label and list of ingredients are important when selecting my dog's food (n=254)**				
Strongly agree	83 (93.3)	135 (81.8)	3.07 (1.23-7.70)	0.017
Neutral	6 (6.7)	30 (18.2)	Ref	
**Good quality extruded dry/kibble or wet/canned pet foods contain all the nutrition my pet needs (n=249)**				
Strongly agree	19 (23.2)	107 (64.1)	0.20 (0.10-0.38)	<0.001
Neutral	36 (43.9)	40 (24.0)	Ref	
Strongly disagree	27 (32.9)	20 (12.0)	1.50 (0.72-3.12)	0.278
**I believe a homemade diet may provide better nutrition for my pet than extruded dry/kibble or wet/canned pet foods (n=232)**				
Strongly agree	41 (50.0)	32 (21.3)	2.07 (1.09-3.93)	0.027
Neutral	31 (37.8)	50 (33.3)	Ref	
Strongly disagree	10 (12.2)	68 (45.3)	0.24 (0.11-0.53)	<0.001
**I believe a raw meat diet may provide better nutrition for my pet than extruded dry/kibble or wet/canned pet foods (n=220)**				
Strongly agree	61 (69.3)	23 (17.4)	5.44 (2.63-11.28)	<0.001
Neutral	19 (21.6)	39 (29.5)	Ref	
Strongly disagree	8 (9.1)	70 (53.0)	0.24 (0.09-0.59)	0.002
**Dogs are carnivores so they need a meat-based diet (n=237)**				
Strongly agree	58 (65.9)	61 (40.9)	3.00 (1.60-5.63)	<0.001
Neutral	19 (21.6)	60 (40.3)	Ref	
Strongly disagree	11 (12.5)	28 (18.8)	1.24 (0.52-2.95)	0.626
**Grains such as wheat, corn, rice, etc. are good sources of nutrition for dogs (n=224)**				
Strongly agree	17 (23.6)	50 (32.9)	0.79 (0.38-1.61)	0.507
Neutral	26 (36.1)	60 (39.5)	Ref	
Strongly disagree	29 (40.3)	42 (27.6)	1.59 (0.82-3.08)	0.167
**Cooking destroys nutrients in pet foods (n=217)**				
Strongly agree	20 (24.1)	26 (19.4)	0.99 (0.48-2.06)	0.976
Neutral	35 (42.2)	45 (33.6)	Ref	
Strongly disagree	28 (33.7)	63 (47.0)	0.57 (0.31-1.07)	0.080
**Dogs need to eat a variety of foods and this should vary from day to day (n=229)**				
Strongly agree	47 (55.3)	27 (18.8)	3.62 (1.86-7.03)	<0.001
Neutral	26 (30.6)	54 (37.5)	Ref	
Strongly disagree	12 (14.1)	63 (43.8)	0.40 (0.18-0.86)	0.019

[*]Confidence intervals (CI) and Odds ratios (OR) refer to the probability of being a member of the raw group; [†]Significance of difference compared with reference (Ref) category

Table S6 Univariate logistic regression analyses evaluating personal nutrition choices and perceived knowledge of human nutrition between dog guardians feeding raw or cooked diets.

	Raw – n (%)	Cooked – n (%)	OR (95% CI)[*]	P-value[†]
**Frequency of thinking about personal nutrition (n=267)**				
Every time you eat	32 (35.6)	82 (46.3)	0.83 (0.40-1.74)	0.626
Regularly but not daily	43 (47.8)	63 (35.6)	1.46 (0.71-3.01)	0.310
Intermittently	15 (16.7)	32 (18.1)	Ref	
**Perceived diet health (n=271)**				
Very healthy	10 (10.9)	24 (13.4)	0.89 (0.37-2.16)	0.793
Moderately healthy	53 (57.6)	83 (46.4)	1.36 (0.74-2.49)	0.318
Neither healthy nor unhealthy	23 (25.0)	49 (27.4)	Ref	
Moderately unhealthy	6 (6.5)	23 (12.8)	0.56 (0.20-1.55)	0.262
**Dietary preference (n=269)**				

Omnivorous	83	(91.2)	154	(86.5)	1.62 (0.70-3.76)	0.264
Non-omnivorous	8	(8.8)	24	(13.5)	Ref	
**Dietary trends/restrictions (n=273)**						
Yes	14	(15.1)	38	(21.1)	0.66 (0.34-1.30)	0.229
No	79	(84.9)	142	(78.9)	Ref	
**Primary individual preparing household meals (n=272)**						
Yes	76	(81.7)	158	(88.3)	0.59 (0.30-1.19)	0.142
No	17	(18.3)	21	(11.7)	Ref	
**Time devoted preparing meals (n=272)**						
Less than 30 minutes	14	(15.1)	25	(14.0)	Ref	
30 minutes to an hour	45	(48.4)	90	(50.3)	0.89 (0.42-1.88)	0.766
Over an hour	34	(36.6)	64	(35.8)	0.95 (0.44-2.06)	0.894
**Meal preparation or batch cooking (n=270)**						
Yes	21	(23.1)	33	(18.4)	1.33 (0.72-2.46)	0.368
No	70	(76.9)	146	(81.6)	Ref	
**Frequency of meal preparation or batch cooking (n=54)**						
≤ 2 days a week	16	(76.2)	21	(63.6)	1.83 (0.54-6.25)	0.336
> 2 days a week	5	(23.8)	12	(36.4)	Ref	
**Frequency of purchasing prepared food (n=263)**						
None this week	20	(22.7)	46	(26.3)	Ref	
1 to 2 times a week	60	(68.2)	101	(57.7)	1.37 (0.74-2.53)	0.319
3 or more times a week	8	(9.1)	28	(16.0)	0.66 (0.26-1.69)	0.384
**Knowledge of human nutrition (n=261)**						
Extreme	12	(13.6)	28	(16.2)	0.97 (0.41-2.30)	0.945
Moderate	57	(64.8)	102	(59.0)	1.27 (0.67-2.37)	0.465
Slight	19	(21.6)	43	(24.9)	Ref	
**Guardians' confidence in making self-directed dietary changes (n=263)**						
Strong	27	(30.7)	51	(29.1)	1.06 (0.48-2.34)	0.888
Moderate	43	(48.9)	87	(49.7)	0.99 (0.47-2.07)	0.976
Neither confident nor unconfident	14	(15.9)	28	(16.0)	Ref	
Unconfident	4	(4.5)	9	(5.1)	0.89 (0.23-3.40)	0.863

*Confidence intervals (CI) and Odds ratios (OR) refer to the probability of being a member of the raw group; †Significance of difference compared with reference (Ref) category

Table S7 Univariate logistic regression analyses examining perceived dog-guardian relationships across guardians feeding raw or cooked diets.

	Raw – n (%)		Cooked – n (%)		OR (95% CI)*	P-value†
**Role of canine in guardian's life: (n=433)**						
**Companion**						
Selected	130	(91.5)	279	(95.9)	0.47 (0.20-1.07)	0.070
Not Selected	12	(8.5)	12	(4.1)	Ref	
**Sports/agility**						
Selected	17	(12.0)	23	(7.9)	1.59 (0.82-3.07)	0.173
Not Selected	125	(88.0)	268	(92.1)	Ref	
**Breeding**						
Selected	8	(5.6)	10	(3.4)	1.68 (0.65-4.35)	0.287
Not Selected	134	(94.4)	281	(96.6)	Ref	

**Obedience**						
Selected	10	(7.0)	12	(4.1)	1.76 (0.74-4.18)	0.199
Not Selected	132	(93.0)	279	(95.9)	Ref	
**Working**						
Selected	9	(6.3)	13	(4.5)	1.45 (0.60-3.47)	0.408
Not Selected	133	(93.7)	278	(95.5)	Ref	
**Showing**						
Selected	7	(4.9)	6	(2.1)	2.46 (0.81-7.47)	0.111
Not Selected	135	(95.1)	285	(97.9)	Ref	
**Perceived dog-guardian relationship (n=420)**						
Companion	13	(9.4)	24	(8.5)	Ref	
Family member	43	(31.2)	97	(34.4)	0.82 (0.38-1.76)	0.607
Owner	24	(17.4)	38	(13.5)	1.17 (0.50-2.72)	0.722
Pet parent	58	(42.0)	123	(43.6)	0.87 (0.41-1.83)	0.715

[*]Confidence intervals (CI) and Odds ratios (OR) refer to the probability of being a member of the raw group; [†]Significance of difference compared with reference (Ref) category
